# Development of a Knowledge Base for an Integrated Older Adult Care Model (SMART System) Based on an Intervention Mapping Framework: Mixed Methods Study

**DOI:** 10.2196/59276

**Published:** 2025-08-14

**Authors:** Rongrong Guo, Shuqin Xiao, Fangyu Yang, Huan Fan, Yanyan Xiao, Xue Yang, Ying Wu

**Affiliations:** 1 School of Nursing, Capital Medical University, 10 You-An-Men Wai Xi Tou Tiao, Fengtai District, Beijing, 100069, China, 86 13910789837; 2 Critical Care Medicine Department, Peking University First Hospital, Beijing, China; 3 Department of Nursing, Beijing Hospital, National Center of Gerontology, Beijing, China

**Keywords:** older adults, home-based care, clinical decision support system, knowledge base, intervention mapping, mHealth

## Abstract

**Background:**

Although mobile health apps integrated with Internet of Things–enabled devices are increasingly used to satisfy the growing needs for home-based older adult care resulting from rapid population aging, their effectiveness is constrained by 3 key challenges: a focus on specific functions rather than on holistic and integrated support, absence of a solid theoretical framework for development, and a lack of personalized, real-time feedback to address diverse care needs. To overcome these limitations, we developed a knowledge-based clinical decision support system using mobile health technology—an intelligent and integrated older adults care model (SMART system).

**Objective:**

This study aims to systematically outline the development process and outcomes of a knowledge base and trigger rules for the SMART system.

**Methods:**

Our study adopted a user-centered approach guided by the nursing process and intervention mapping (IM) framework. We first identified older adult care needs through semistructured, in-depth interviews. Guided by the nursing process and informed by guidance from the World Health Organization’s Integrated Care for Older People and World Health Organization International Classification of Functioning, Disability, and Health, along with the North American Nursing Diagnosis Association-I nursing diagnosis, we then determined care problems along with their underlying causes and risk factors and diagnostic criteria. Building on these findings, we applied the first 3 steps of the intervention mapping framework to formulate corresponding long-term and short-term care objectives, select appropriate evidence-based interventions, and match practical implementation approaches, which were grounded in rigorous evidence derived from systematic literature reviews, clinical guidelines, and expert insights. We also developed a set of trigger rules to link abnormalities in older adults with corresponding care problems and interventions in the SMART knowledge base.

**Results:**

The semistructured in-depth interviews identified 5 types of care needs—daily life care, health care, external support, social participation, and self-development—which formed the foundation of the SMART knowledge base. Based on this, we identified 138 care problems, each with associated causes and risk factors and diagnostic criteria. The objective matrix comprised 138 long-term and 195 short-term care objectives. Guided by 15 expert-defined selection criteria, we then selected 450 evidence-based interventions, each paired with at least 1 feasible and practical implementation approach. Additionally, we developed diagnostic rules to match the assessment data with relevant care problems and their causes and risk factors and intervention trigger rules to formulate personalized interventions based on individual characteristics, ensuring tailored care aligned with specific care objectives.

**Conclusions:**

This study outlines the development process and outcomes of the SMART knowledge base and trigger rules. The study methodology offers theoretical support for developing knowledge bases and trigger rules of similar clinical decision support systems for home-based older adult care.

## Introduction

As populations age rapidly worldwide, a growing number of older adults are experiencing physiological decline and various health issues [1]. This demographic shift necessitates increased daily assistance and medical care to ensure optimal quality of life, thereby exerting substantial pressure on global health and social care systems [2]. In China, home to the largest older adult population globally, the burden of older adult care is particularly pronounced [3]. The country has adopted the “9073” model, in which 90% of older adults are cared for in their own homes, 7% rely on community services, and 3% receive institutional care [4]. However, this model faces substantial challenges, as the one-child policy and the rising labor mobility from urbanization have eroded traditional family-based care structures [5]. At the same time, older adult care services are often fragmented and inconsistent, involving multiple providers across different institutions. These issues, combined with a shortage of professional care providers, have resulted in a suboptimal home-based older adult care model that struggles to meet the complex needs of older adults [6,7]. As a result, informal caregivers are burdened with heightened stress, emotional strain, and financial pressure as they need to coordinate care, manage complex health needs, and navigate various health care systems [8]. At a societal level, these systemic inefficiencies can lead to redundant treatments, delayed interventions, and resource inefficiency, ultimately elevating health care costs and threatening economic sustainability [9,10].

A proactive older adult care model emphasizes continuous monitoring of older adults’ health and living conditions, allowing early detection of health changes and timely identification of care needs. This approach supports prompt and targeted interventions to prevent health deterioration. However, traditional monitoring methods, such as routine home visits, periodic paper-based assessments, and manual health checks, are often impractical due to the dispersed living situations of older adults and the severe shortage of older adult care resources [11]. The emergence of mobile health (mHealth) technology, in conjunction with the Internet of Things, offers a promising alternative to traditional monitoring methods. mHealth apps, often supported by Internet of Things–enabled devices such as wearable sensors and remote monitors, are increasingly used in older adult care for communication, real-time tracking, education, and chronic disease management, providing cost-effective and scalable solutions unrestricted by time or location [12,13].

While these technologies hold great potential to transform older adult care, their current apps remain limited by several challenges. One major challenge is the lack of a comprehensive understanding of older adults' diverse and evolving care needs. As a result, many apps tend to focus narrowly on specific functions such as daily care or medication reminders, rather than delivering integrated and holistic support [14,15]. Another challenge is that many apps were developed without a structured and theory-driven framework, resulting in poorly supported evidence for their effectiveness [16,17]. Furthermore, given the diversity and complexity of older adults’ health status and characteristics, such as variations in chronic conditions, functional abilities, and care preferences, personalized interventions are essential to ensure that care strategies are tailored to each individual’s unique needs. Real-time feedback is also critical for promptly detecting health fluctuations, enabling timely adjustments to care plans, and preventing minor issues from developing into serious complications. Unfortunately, most existing apps lack these features, making them less effective compared to interactive apps that support personalized care and timely decision-making [18].

To address the limitation of current mHealth apps being confined to specific functions, a user-centered design approach offers a promising solution. Unlike traditional software-centric design, user-centered design emphasizes the perspectives and experiences of older adults, thus enabling a deeper understanding of their multifaceted care needs [19]. Additionally, the Continuity Assessment Record and Evaluation (CARE) tool (Multimedia Appendix 1), developed by the Centers for Medicare & Medicaid Services, can serve as a tool to ensure continuous, consistent, and comprehensive assessments of older adults by measuring their medical, functional, cognitive, and social support factors. Its multidimensional structure enables a holistic understanding of individual care needs, supporting integrated older adults care plans. The Chinese version of the CARE tool has demonstrated good reliability and validity, particularly in stroke patients, reinforcing its applicability in home-based older adult care [20].

A promising solution to the lack of a structured and theory-driven framework in many mHealth apps lies in the use of the intervention mapping (IM) framework. This systematic approach guides the development of behavior change interventions through six sequential steps: (1) conducting a needs assessment and building a logic model, (2) specifying change objectives, (3) selecting theory-informed methods and practical strategies, (4) designing the intervention program, (5) planning for adoption and implementation, and (6) developing an evaluation strategy [21,22]. Grounded in social and behavioral science theories, IM has been proven effective in various public health contexts, especially in chronic disease self-management [23]. Its structured nature and theoretical grounding make it well-suited for determining interventions that respond to the complex care needs of older adults living at home.

Regarding the limitation of lacking personalization and real-time feedback, clinical decision support systems (CDSSs) present a more reliable option by linking individual health data with medical knowledge [24]. CDSSs are technology-driven systems to enhance health care delivery by facilitating medical decisions through the use of targeted clinical knowledge and patient data. Based on decision-making mechanisms, CDSSs are categorized into data-based and knowledge-based systems [25]. While some data-based CDSSs benefit from advances in explainable artificial intelligence (AI), they are still often considered “black-box” models, where the reasoning processes are difficult to trace. This opacity can result in inaccurate or conflicting recommendations that may deviate from the established guidelines and even jeopardize the health of older adults [26]. In contrast, knowledge-based CDSSs provide more transparent decision-making support by correlating individual data with relevant information in a knowledge base through a reasoning module known as an inference engine [27]. The inference engine uses a set of predefined rules and algorithms to analyze the collected data, drawing on the preset knowledge items in the corresponding knowledge base to generate transparent and evidence-based recommendations that assist clinicians in making informed decisions.

Therefore, leveraging mHealth technology, we have developed a knowledge-based CDSS for proactive home-based older adults care—an Intelligent and Integrated Older Adults Care Model. Similar to how the neural reflex functions in biological systems, the model functions as the “neural reflex” for older adults care, which includes Sensors and Scales (serves as the receptor), a mobile phone autonomous response system for the app (serves as the nervous system in the spinal cord), a remote cloud and management center (serves as the central nervous system in the brain), and total care system (serves as the effector, where various care providers are incorporated for specific types of care services for older adults), collectively referred to as the SMART system. The Sensors and Scales component collected integrated data from older adults via periodic CARE self-assessments and wearable devices. These data were automatically uploaded to the Remote Cloud and Management Center via Wi-Fi or 5G networks for comprehensive analysis to detect abnormalities and diagnose current or potential care problems using preconfigured knowledge items and trigger rules. Based on the diagnostic results, and considering each individual’s characteristics and preferences, the Remote Cloud and Management Center can formulate tailored interventions, which are then appropriately distributed to daily caregivers (eg, family members and nannies) or professional care providers (eg, doctors, nurses, and domestic workers) within the Total Care System. The Mobile Phone Autonomous Response System was a set of simple algorithms to deal with simple but urgent care problems, similar to the coordinated reflex actions within the spinal cord, such as the withdrawal reflex from a painful stimulus. The entire process targets 12 key domains for older adults: decreased or lost self-care ability, falls, impaired skin integrity, delirium, dysphagia, urinary retention, constipation, common diseases, incontinence, cognitive decline, depression, and psychosocial issues.

This paper aims to outline the development of the SMART system’s knowledge base and trigger rules guided by a qualitative, user-centered approach to determine older adults’ care needs. The development process followed the nursing process and the IM framework. Additionally, the paper presents the results of this innovative knowledge base.

## Methods

### Overview

The nursing process and IM framework jointly guided the development and maintenance of our SMART knowledge base and trigger rules. Guided by the nursing process, we first determined care problems along with their underlying causes and risk factors and diagnostic criteria. Building on these findings, we applied the first 3 steps of the IM framework to formulate corresponding long-term and short-term care objectives, select appropriate evidence-based interventions, and match practical implementation approaches. Steps 4‐6 of the IM framework, which pertain to program production, implementation, and evaluation, were used to guide the design, development, and evaluation of the SMART system and have been reported elsewhere [28].

A multidisciplinary team with extensive experience in gerontological care, cognitive and socio-psychological care, cardiovascular care, delirium care, and emergency care collaborated to develop the SMART knowledge base and trigger rules. The core development of the SMART knowledge base and trigger rules was conducted between December 2015 and December 2016. Due to significant changes in economic conditions, technological advancements, and shifts in the health care landscape, some aspects of home-based older adult care have evolved, rendering previous practices less relevant and introducing new interventions. To ensure the knowledge base remains up-to-date and aligned with current older adult care priorities, our research team conducted maintenance and updates of the SMART knowledge base and trigger rules between October 2024 and March 2025 to ensure its scientific rigor, relevance, and logical consistency.

We reported the development process in accordance with the Template for Intervention Description and Replication (TIDieR) checklist [29].

### Ethical Considerations

The study was approved by the Institutional Review Committee of the Capital Medical University (approval numbers 2015SY49U and 2020SY047). Before participating in the study, we provided all potential participants with a comprehensive explanation of the study's objectives, methods, procedures, and the data to be collected. Written informed consent was obtained from each participant before their enrollment in the study. Participants were assured of their right to withdraw from the study at any time without any penalties or adverse consequences. To safeguard participants' privacy and confidentiality, all personal identifiers were securely stored in password-protected files. The analysis and reporting of study findings used only deidentified or anonymized data, ensuring that participants' identities remained confidential.

### Step 1: Needs Assessment of Older Adults and Their Caregivers

We conducted needs assessments using semi-structured in-depth interviews to explore home-based care needs and preferences regarding system functionality (eg, reminders) and the user interface of the SMART system for older adults and their caregivers. We also assessed their preferences for system workflow, information presentation formats, and intervention delivery methods (eg, text, voice, or video).

After obtaining ethical approval, trained interviewers conducted 2 rounds of face-to-face, semistructured, in-depth interviews. The first round took place in Guangnei Street, Xicheng District, Beijing, between December 2015 and March 2016 (approval no. 2015SY49), and the second round was carried out in Youzhu Community, Fengtai District, Beijing, between October 2024 and November 2024 (approval no. 2020SY047). In both rounds, purposive sampling was used to recruit adults aged 60 years and older who had lived in the respective districts for at least 6 months if they had either been diagnosed with at least 1 noncommunicable disease or experienced physical or cognitive disabilities of different severity. Older adults were excluded if they (1) were unable to speak or understand Mandarin, (2) had severe bilateral hearing impairment, or (3) refused to participate.

After explaining the study purpose and interview process to each participant or their surrogate, interviewers conducted the interviews following a predefined guide (File 1 in Multimedia Appendix 2) in participants’ homes, a relatively quiet and familiar setting, until theoretical saturation was reached. Theoretical saturation was defined as the interviewers agreed no new information was generated from new participants. The interview guide consisted of several open-ended questions initially drafted by the interviewers and subsequently refined by the broader research team. All interviews were recorded in both written and audio formats, with informed consent obtained from the participants or their surrogates.

### Step 2: Determining Care Problems, Causes and Risk Factors, and Diagnostic Criteria

In the initial core development of the SMART knowledge base, we followed the guidance of the World Health Organization’s (WHO’s) Integrated Care for Older People (ICOPE) and the WHO International Classification of Functioning, Disability, and Health (ICF), along with the North American Nursing Diagnosis Association (NANDA)-I (2015‐2017) nursing diagnosis framework to identify care problems and their causes and risk factors. During the maintenance phase of the SMART knowledge base, in light of the recent updates to the NANDA-I framework, we compared the NANDA-I (2015‐2017) and NANDA-I (2024‐2026) editions to identify discrepancies. Based on this analysis, we revised the identified care problems, introduced new care problems, and accordingly updated the associated causes and risk factors and diagnostic criteria.

The ICOPE guideline provides a proactive model for older adult care, emphasizing both functional ability and individual needs [30]. The ICF framework helps evaluate levels of functioning and disability among older adults by considering the interaction between health conditions, environmental factors, and personal attributes [31]. Meanwhile, the NANDA-I framework offers standardized terminology and supports the determination of diagnostic criteria for care problems [32]. Diagnostic criteria for the care problems were determined based on the NANDA-I.

Since the main goal of this study was to develop a knowledge base and trigger rules that could support the SMART system, we only included a limited set of common diseases, including hypertension, coronary heart disease, diabetes mellitus, and pneumonia, to reduce workload and ensure feasibility in the development of the SMART knowledge base [33].

### Step 3: Development of Evidence-Based Interventions, Implementation Approaches, and Trigger Rules

#### Generation of Care Objectives Corresponding to the Care Problems

During both the initial development and subsequent maintenance of the SMART knowledge base, we formulated long-term and short-term care objectives instead of performance objectives and change objectives suggested by the IM framework. This is because the IM is primarily used to guide the development, implementation, and evaluation of behavioral change, while our interventions were aimed at solving older adults’ care problems. The long-term care objectives represent the expected outcomes after providing care services, while the short-term care objectives are periodic objectives that will collectively achieve the long-term care objectives.

#### Determination of Criteria for the Selection of Evidence-Based Interventions

A set of criteria for selecting potentially effective evidence-based interventions for older adults is of vital importance [34]. Based on the IM framework, we determined 2 types of criteria for both the initial development and maintenance phases of the SMART knowledge base: general criteria, the most basic and necessary criteria to identify the existing effective interventions that are appropriate for older adults, and desirable criteria, all other criteria to ensure interventions are practical, safe, and helpful for older adults cared for at home [35].

#### Review of the Existing Evidence-Based Interventions

During the initial development and subsequent maintenance of the SMART knowledge base, we conducted 2 systematic literature reviews to identify relevant studies and guidelines on evidence-based interventions for the identified care problems by searching the PubMed database thoroughly. The first review focused on publications before December 2016, when we began developing the SMART knowledge base. The second supplementary review was conducted between December 2024 and February 2025, primarily targeting new literature and guidelines from December 2024 to March 2025. Both reviews followed the same search strategy, with the detailed methods summarized in File 2 of Multimedia Appendix 2. We did not restrict the search by publication type or language. Reports on randomized controlled trials (RCTs), non-RCTs, and relevant guidelines were all included. To assess the quality and effectiveness of the interventions, we used the 5-point Jadad scale [36] for RCTs and the UK Cochrane Center Evidence Level (2001) for non-RCTs.

#### Selection of the Evidence-Based Interventions

During the initial development of the SMART knowledge base, the research team selected potentially effective evidence-based interventions after multiple discussions according to the predefined general and desirable criteria. In total, 5 researchers independently screened for interventions from the identified guidelines and literature according to the general criteria, excluding those that did not meet the requirements. Subsequently, each researcher independently ranked the remaining interventions against the desirable criteria. Final decisions were made through team discussions until consensus was reached. Similarly, during the maintenance phase of the SMART knowledge base, 4 researchers followed the same process to screen and rank new evidence-based interventions based on newly identified guidelines and literature.

The identified care problems, associated causes and risk factors, corresponding diagnostic criteria, and the drafted evidence-based interventions were confirmed through consultations with a multidisciplinary expert panel (MEP). Specifically, 2 rounds of expert consultation (rounds 1 and 2) were conducted during the initial development phase, while an additional round (round 4) was carried out during the maintenance phase on March 6, 2025. The composition of the MEP remained consistent throughout the process, comprising 2 gerontological care experts, 1 geriatric physician, and 1 geriatric nurse. These consultations also provided valuable recommendations on other potentially effective interventions and critical considerations for developing the SMART system.

#### Development of Intervention Categories

Most older adults have multiple care problems, and interventions targeting different care problems might have the same methods (eg, diet and nutrition). However, the specific requirements of interventions might be different or even conflicting (eg, a high-protein diet for severe anemia and a high-quality, low-protein diet for chronic renal failure). To address this, we created intervention categories (eg, health education, diet, and nutrition) that allow us to assign each intervention into 1 category only. After the SMART system automatically develops an integrated care plan, this approach allows us to combine similar interventions and examine whether there are different or even conflicting interventions for the same intervention category (eg, diet, exercise), and to select the most appropriate interventions for older adults according to their holistic conditions. The intervention categories were applied throughout the initial development and subsequent maintenance of the SMART knowledge base.

#### Translation From Evidence-Based Interventions Into Implementation Approaches

To improve the accuracy and efficiency of intervention delivery, we combined the characteristics, preferences, and ambient conditions of the care providers to translate evidence-based interventions into practical implementation approaches by clearly presenting what (content to deliver or methods to deliver the intervention), when (time to deliver), how many (dose), how often (frequency), who (the person who will implement the interventions), and by what types of presentation format for information sent to the care providers [37]. To make the implementation approaches clearer and easier to understand, the presentation format varies among care providers and situations. For example, interventions targeting older adults, family members, and informal care providers will be delivered in the form of pictures, comics, voice, and video. In contrast, interventions targeting professional nursing staff will mainly be provided by text and pictures. This process applies to both the initial development and subsequent maintenance phases of the SMART knowledge base.

#### Formulation of Trigger Rules

Considered as one of the most widely used reasoning methods in knowledge-based CDSSs, IF-THEN rules were used within the SMART system’s inference engine to enable reasoning and trigger relevant implementation approaches. These rules are categorized into diagnostic rules and intervention trigger rules. The diagnostic rules match the assessment data (eg, the older adults’ abnormal conditions) with the determined care problems and their causes and risk factors, while the intervention trigger rules ensure that older adults receive personalized interventions tailored to their characteristics (eg, demographic features, medical conditions, functional status, cognitive status, and preferences) to achieve the care objectives developed based on the identified care problems and their causes and risk factors. The inference engine processes these rules by continuously evaluating the collected data, dynamically diagnosing accurate care problems, and appropriately matching the most appropriate interventions, ensuring timely delivery and feedback based on real-time patient conditions.

During the initial development of the SMART knowledge base, the accuracy and feasibility of the implementation approaches and trigger rules were assessed through a third round of consultation with the MEP (round 3). In the maintenance phase, a fifth round of expert consultation (round 5) was conducted on March 25, 2025, to evaluate the accuracy and feasibility of the newly revised implementation approaches and trigger rules.

## Results

### Needs of Older Adults

A total of 26 older adults were interviewed, with 21 in the first round and an additional 5 participants in the second round. The average age was 79.15 (SD 7.78) years old, with 46.15% (n=12) being male. The detailed baseline characteristics of the interviewees are summarized in File 3 of Multimedia Appendix 2. Since this paper aims to report the development process and results of the SMART knowledge base and trigger rules, only the care needs related to the intervention contents for older adults are presented. The identified care needs include 5 types: daily life care, health care, external support, social participation, and self-development. The first 4 types were identified in the first round of interviews, while the self-development needs, identified in the second round, specifically emphasize the need for digital inclusion and technology use training. Full details of the interview results are presented in File 4 of Multimedia Appendix 2.

### Care Problems, Causes and Risk Factors, and Diagnostic Criteria

During the initial development process of the SMART knowledge base, we identified 11 core domains of care problems in alignment with the ICOPE guidelines, ICF, and NANDA-I (2015‐2017), while incorporating insights from needs assessments and considering the distinctive characteristics, preferences, and environmental conditions of older adults. These domains include decreased or lost self-care ability, falls, delirium, dysphagia, incontinence, constipation, urinary retention, cognitive decline, depression, impaired skin integrity, and common diseases. We further divided each domain into 2 subdomains: risk for care problems and presence of care problems. Through collaborative discussions, we identified a total of 137 care problems, along with their causes and risk factors, from these 11 domains. Following the first 2 rounds of consultations with the MEP, an additional care problem was identified: risk for delayed detection of delirium causes. Consequently, the initial knowledge base comprises a total of 138 care problems.

In the subsequent maintenance phase of the SMART knowledge base, we identified a new core domain of care problems: psychosocial issues. Within this domain, we recognized 3 additional care problems: loneliness, social isolation, and digital divide. Additionally, we refined the descriptions of the previously identified 138 care problems. The final compilation encompasses 141 care problems systematically categorized under the 12 core domains and subdomains, with detailed information available in File 5 of Multimedia Appendix 2. Table 1 presents examples of nursing issues, including their classification and associated causes and risk factors.

**Table 1.** Matrices of care problems and their subdomains, associated causes and risk factors, and diagnostic criteria related to the impaired skin integrity domain: examples.

Care problems	Subdomains of care problems	Causes and risk factors	Diagnostic criteria
Risk for pressure ulcers, related to prolonged periods of unrelieved pressure	Risk for impaired skin integrity	Prolonged periods of unrelieved pressure due to extended bed rest	Older adults are recorded not rolling left and right for more than 4 hours AND score 1^a^/2^b^/3^c^ on CARE^d^ item VI-C3^e^
Risk for diabetic foot ulcers	Risk for impaired skin integrity	DM^f^ with loss or decrease of sensation in the lower extremities	Older adults experience difficulties from DM (CARE item III-B) AND have weakened sensation in their lower extremities
Impaired skin integrity: Stage 3 pressure ulcers	Presence of impaired skin integrity	—^g^	Older adults score 1^h^ on CARE item III-G2^i^ AND score 1/2/3/4/5/6/7/8^j^ on CARE item III-G2b^k^

a 1 score for VI-C3: dependent.

b 2 scores for VI-C3: Substantial or maximal assistance.

c 3 scores for VI-C3: Partial or moderate assistance.

d CARE: Continuity Assessment Record and Evaluation.

e VI-C3: the ability to roll from lying on back to left and right side and roll back to back.

f DM: diabetes mellitus.

g Not applicable.

h 1 score for III-G2: this patient has one or more unhealed pressure ulcer(s) at stage 2 or higher or unstageable.

i III-G2: does this patient have one or more unhealed pressure ulcer(s) at stage 2 or higher or unstageable?

j 1/2/3/4/5/6/7/8 for III-G2b: the number of stage 3 pressure ulcers is 1/2/3/4/5/6/7/8.

k III-G2b: please specify the number of pressure ulcers at stage 3.

### Evidence-Based Interventions, Implementation Approaches, and Trigger Rules

#### Care Objectives Corresponding to the Care Problems

During the initial development phase of the SMART knowledge base, we conducted a thorough analysis of the identified 138 care problems and their causes and risk factors, establishing 138 long-term and 195 short-term care objectives. Additionally, in response to 3 care problems identified in the maintenance phase, we formulated 3 long-term and 6 short-term care objectives. As a result, the final SMART knowledge base comprises 141 long-term and 201 short-term care objectives. These objectives align closely with the primary goal of our SMART system: to reduce physical or cognitive disabilities, preserve physical and cognitive capacities to the fullest extent, and promote rehabilitation to mitigate functional decline in older adults. To facilitate implementation, we categorized the care objectives into 2 groups: those targeting prevention and early detection and those directed toward prompt intervention and early rehabilitation. Table 2 illustrates the care objectives matched to the identified care problems and their corresponding causes and risk factors.

**Table 2.** Matrices of care objectives matched to the identified care problems and their corresponding causes and risk factors related to the impaired skin integrity domain: examples.

Care problems	Causes and risk factors	Categories of care objectives	Long-term care objectives	Short-term care objectives
Risk for pressure ulcers, related to prolonged periods of unrelieved pressure	Prolonged periods of unrelieved pressure due to extended bed rest	Prevention and early detection of impaired skin integrity	Maintain skin integrity	Reduce pressure time at the bony prominence
Risk for diabetic foot ulcers	DM^a^ with loss or decrease of sensation in lower extremities	Prevention and early detection of impaired skin integrity	Avoid diabetic foot	Avoid damage to foot skin
Impaired skin integrity: stage 3 pressure ulcers	—^b^	Prompt intervention and early rehabilitation of impaired skin integrity	Facilitate the healing of pressure ulcers through dedicated care	Prevent the advancement of pressure ulcers to stage 4

a DM: diabetes mellitus.

b Not applicable.

#### Criteria for the Selection of Evidence-Based Interventions

As detailed in Textbox 1, during the initial development phase of the SMART knowledge base, we formulated 2 general criteria and 13 desirable criteria to guide the selection of potentially effective evidence-based interventions for older adult care. These criteria were also used in the subsequent maintenance phase of the SMART knowledge base. The general criteria focus on interventions that directly address the identified care problems and care objectives, tailored to the specific causes and risk factors of these care problems. The desirable criteria encompass factors such as availability, safety, effectiveness, integration, personalization, simplicity, and affordability.

Textbox 1. **Criteria for selecting evidence-based interventions for older adult care.**

**General criteria:**

- Related to the care problems and short-term care objectives
- Designed for the causes and/or risk factors corresponding to the care problems

**Desirable criteria:**

- Effectiveness indicated by a high odds ratio from the existing literature
- Recommended in existing guidelines
- Recognized by health professionals in the field of aging
- Easy to deliver in a home context
- Safe for the target older adults
- Culturally appropriate
- Able to integrate into older adults’ daily life and habits
- Simple and straightforward to understand
- Easy to implement
- Personalized
- With appropriate frequency and dose
- With positive feedback from older adults shown in existing literature
- Time-saving, labor-saving, and money-saving

#### Evidence-Based Interventions and Intervention Categories

After systematically searching the PubMed database using established strategies, our research team identified relevant literature and guidelines and accessed their full texts from appropriate databases. A subsequent quality assessment revealed that the majority of the retrieved literature was of low quality. As a result, we primarily incorporated relevant guidelines to derive evidence-based interventions. Specifically, during the initial development phase of the SMART knowledge base, the research team drafted a total of 446 evidence-based interventions that satisfied our selection criteria after 9 rounds of discussion. In total, 2 rounds of consultation with the MEP (File 6 of Multimedia Appendix 2) led to the inclusion of 4 more evidence-based interventions, bringing the total to 450.

During the maintenance phase, the research team added 7 new evidence-based interventions, which were subsequently confirmed through a round of consultation with the MEP. As a result, the final SMART knowledge base comprises a total of 457 evidence-based interventions. Table 3 provides information about these 7 new interventions in detail.

**Table 3.** The 7 new evidence-based interventions identified in the maintenance phase.

Domains of care problems	Evidence-based interventions
Falls	Home environment modifications: Install grab bars, non-slip flooring, and remove tripping hazards after a safety audit
Cognitive decline	Use digital cognitive training platforms for daily mental exercise
Common diseases	Use smart pill dispensers to improve medication adherence
Psychosocial issues	Introduce AI-powered^a^ social robots to provide companionship, reminders, and friendly dialogue Facilitate participation in web-based or face-to-face interest groups Provide structured, age-friendly training on using smartphones, tablets, and health apps Organize intergenerational programs linking youth and older adults both online and in person

a AI: artificial intelligence.

Based on the finalized 457 evidence-based interventions, we created 13 intervention categories: health education, diet and nutrition, rest and exercise, monitoring of physiological function and health status, addressing basic living and safety needs, skin cleaning and hygiene, functional exercise, rehabilitation, implementation of nursing and treatment procedures, medication management, social support, psychological support, and lifestyle monitoring. We then classified the determined interventions into these 13 categories according to their characteristics, target population, mode of delivery, and purpose.

#### Implementation Approaches and Trigger Rules of the Evidence-Based Interventions

For each determined evidence-based intervention, we developed at least 1 feasible and practical implementation approach suitable for specific contexts. We further validated the feasibility of these approaches during the third consultation with the MEP in the development phase and the fifth consultation with the MEP during the maintenance phase of the SMART knowledge base. Table 4 presents examples of care problems, evidence-based interventions, intervention categories, and implementation approaches related to the impaired skin integrity domain.

**Table 4.** Matrices of care problems, evidence-based interventions, intervention categories, and implementation approaches related to the impaired skin integrity domain: examples.

Care problems	Interventions	Intervention categories	Intervention trigger rules (IF)	THEN: implementation approaches					
				What	Who	How many	How often	When	By what types of presentation format
Risk for pressure ulcers, related to prolonged periods of unrelieved pressure	Frequent repositioning	Rest and exercise	Older adults score 1^a^/2^b^/3^c^ on CARE^d^ item VI-C3^e^ AND remain the same position for ≥4 hours	Mr. Li has been in the same position for over 4 hours, increasing his risk of pressure ulcers. Regular turning can prevent sustained pressure and the onset of ulcers. Please assist Mr. Li in changing positions now, and fill out the turning record form in the SMART system.	Family members or primary caregivers	—^f^	Once every 2 hours	Every 2 hours after the initial trigger	Text and tutorial video
Risk for diabetic foot ulcers	Knowledge tips for foot skin protection	Health education	Older adults score 13‐15^g^ on CARE items IV-B3a, IV-B3b, and IV-B3c^h^ AND score 3 on CARE item V-C1c^i^	Mr. Li, please check between your toes for ulcers or discoloration. Also, please take a photo and upload it to the SMART system so that we can detect any abnormality of your foot skin in time.	Older adults	—	Once a day	7 PM daily	Text
Impaired skin integrity: Stage 3 pressure ulcers	Wound care: cleansing	Skin cleaning and hygiene	Older adults are rated as E3d^j^ or E4d^k^ on CARE item II-E1d	Mr. Li has developed stage 3 pressure ulcers and is prone to infection. Please take Mr. Li to a professional hospital department and consult an experienced nurse for treatment.	Family members or primary caregivers	—	—	Once evaluated	Text

a 1 score for VI-C3: dependent.

b 2 scores for VI-C3: Substantial or maximal assistance.

c 3 scores for VI-C3: Partial or moderate assistance.

d CARE, Continuity Assessment Record, and Evaluation.

e VI-C3: the ability to roll from lying on back to left and right side and roll back to back.

f Not applicable.

g 13‐15 on IV-B3a, IV-B3b, and IV-B3c: normal cognitive function.

h IV-B3a, IV-B3b, and IV-B3c: brief interview for mental status.

i 3 scores for V-C1c: ability to see in adequate light is adequate (see fine detail, including regular print in newspapers or books).

j E3d on II-E1d: Caregivers will need training and other supportive services in medical procedures/treatments.

k E4d on II-E1d: Caregivers not likely to be able in medical procedures or treatments.

Each implementation approach operates on a set of IF-THEN rules, targeting either older adults, their primary caregivers, or medical staff, depending on who will implement the intervention. Consider the case of an older adult, Mr. Li. *IF* Mr. Li is identified with the care problem “risk for pressure ulcers, related to prolonged periods of unrelieved pressure” due to prolonged bed rest, *THEN* the personalized intervention involves prompting frequent repositioning, which falls under the intervention category of rest and exercise. For instance, *IF* Mr. Li cannot independently reposition and the SMART system has not received a repositioning record for over 4 hours, *THEN* the SMART system automatically sends a message (what) to his primary caregiver (who) every 2 hours (how often), using a standardized text format (by what types of presentation format). The message reads: “Mr. Li has been in the same position for over four hours, increasing his risk of pressure ulcers. Regular turning can prevent sustained pressure and the onset of ulcers. Please assist Mr. Li in changing positions now, and fill out the turning record form in the SMART system.”

## Discussion

### Principal Findings

This paper outlines the systematic development and outcomes of the knowledge base and trigger rules for our SMART system, a comprehensive and integrated care model designed to proactively deliver home-based care for older adults. The knowledge base, developed in accordance with the nursing process and IM framework, incorporates interventions tailored to older adults’ specific care needs. These interventions are grounded in the latest literature, guidelines, and expert opinions. To the best of our knowledge, this study represents the first exploration of a knowledge-based CDSS in the realm of integrated home-based care for older adults. Our meticulous methodology provides a theoretical foundation for the future development of knowledge bases in CDSSs aimed at supporting the delivery of home-based care for this population.

Contemporary knowledge-based CDSSs predominantly focus on disease diagnosis in clinical settings [38], necessitating extensive knowledge from diverse fields and intricate operating rules to function effectively [39]. However, the vastness, complexity, and rapid evolution of medical knowledge pose significant challenges in developing and maintaining these knowledge bases as well as the corresponding rules [40,41]. Our SMART system, however, stands out for practical feasibility, as it concentrates on delivering home-based care for older adults. This focus entails a more limited scope of professional knowledge, thereby significantly reducing the costs associated with both development and upkeep.

The SMART knowledge base was initially developed between December 2015 and December 2016, before the widespread adoption of many transformative digital health technologies. At that time, tools such as AI-powered CDSSs, Food and Drug Administration–approved consumer-grade wearable devices, integrated telehealth platforms with social engagement features, and large language models were either in their early stages or unavailable. Since then, both the care needs of older adults living at home and the field of home-based older adult care have evolved significantly. Previously effective interventions may no longer be suitable, while new evidence-based strategies have emerged to address evolving priorities in older adult care.

To ensure the SMART knowledge base remains aligned with current priorities, a comprehensive maintenance was conducted between October 2024 and March 2025. Following renewed ethical approval in 2020, we expanded semistructured in-depth interviews with older adults and their primary caregivers to explore emerging care needs from October to November 2024. In updating care problems, associated causes and risk factors, and diagnostic criteria, we adopted the NANDA-I (2024‐2026) guidelines instead of the 2015‐2017 edition. A supplementary review of the latest guidelines and studies (December 2024- February 2025) identified emerging evidence-based interventions and flagged outdated ones, which were thoroughly discussed during an expert consultation on March 6, 2025. A final consultation on March 25, 2025, ensured the updated implementation approaches and trigger rules reflected current best practices.

Notably, our extended interviews revealed a self-development need among older adults, particularly related to digital inclusion and technology use training. This finding underscores a growing trend of accelerated technology adoption and affirms the potential of our system to support older adults in engaging with digital tools to some extent. In addition, several entries in the SMART knowledge base were updated to reflect advancements in information technology and large language models, including digital cognitive training platforms for mental exercise, smart pill dispensers to improve medication adherence, and AI-powered social robots for companionship and reminders.

A key strength of this study is the adoption of the user-centered design. In contrast to many apps for older adults that rely on software-centric design and often overlook users’ actual care needs [42,43], the user-centered interview approach enables a comprehensive understanding of the multifaceted care needs of older adults. This approach enhances the precision and relevance of the recommended interventions, promotes comprehensive care management, and improves overall health outcomes, while also fostering greater trust in the SMART model among older adult individuals [44,45].

The systematic development of evidence-based interventions in our SMART knowledge base represents another notable strength. Although the range of interventions targeting older adults is expanding, many lack a structured and systematic framework for development and are often supported by limited or insufficient evidence [46]. To enhance the potential effectiveness of these interventions, we amalgamated comprehensive insights from user-centered interviews and an exhaustive literature review to identify relevant care problems, guided by ICOPE, ICF, and NANDA-I guidelines. Following the IM framework, we thoroughly reviewed the latest literature and guidelines to formulate evidence-based interventions [47]. Furthermore, our iterative refinement process, including 4 rounds of expert consultations, allowed us to adjust the interventions from a professional perspective, thereby enhancing both the effectiveness and the practical applicability of our SMART knowledge base [48].

However, this study has several limitations. First, excluding professional medical staff from the interviews may have resulted in overlooking certain care needs [49]. Although this gap was partially addressed through consultations with the MEP, the potential for oversight remains. Second, we primarily included individuals who were cognitively intact and willing to participate, which may have introduced survivorship bias. Older adults with cognitive limitations often have more complex and diverse care needs, and their perspectives may be underrepresented in our findings. To address this, we interviewed family members or primary caregivers of older adults who could not communicate and also reviewed relevant literature and guidelines to ensure a comprehensive understanding of care needs. Third, our literature review was confined to the PubMed database, which, despite being a comprehensive biomedical literature resource, might have led to omissions of valuable information [50]. Fourth, in the common disease category, our focus was restricted to major health-impacting conditions, such as hypertension, diabetes mellitus, coronary heart disease, and pneumonia. While this focus aligns with the aim of supporting home-based older adult care, it inevitably narrows the scope of disease management. Fifth, although the knowledge base was updated between October 2024 and March 2025, new evidence may still emerge that has not yet been incorporated. We will continue maintaining the knowledge base and explore the integration of large language models with knowledge graphs to accelerate the updating process. Lastly, although grounded in robust literature evidence and expert insights following a theoretical framework, the real-world usefulness of the SMART knowledge base remains unverified, as the acceptability, feasibility, and effectiveness of its interventions have not been empirically validated. An RCT is planned to evaluate these aspects, and the results will be disseminated in future publications.

### Conclusions

This article presents the development and outcomes of the knowledge base and trigger rules of the SMART system. The study methodology provides theoretical support for further development of knowledge bases in CDSSs aimed at delivering home-based older adults care.

## Supplementary material

10.2196/59276 Multimedia Appendix 1 CARE tool.

10.2196/59276 Multimedia Appendix 2 Detailed methods and results of interviews and expert consultations.

## Abbreviations

AI: artificial intelligence
CARE: Continuity Assessment Record and Evaluation
CDSS: clinical decision support system
ICF: International Classification of Functioning, Disability, and Health
ICOPE: Integrated Care for Older People
IM: intervention mapping
MEP: multidisciplinary expert panel
mHealth: mobile health
NANDA: North American Nursing Diagnosis Association
RCT: randomized controlled trial
TIDieR: Template for Intervention Description and Replication
WHO: World Health Organization
