# Supplementary figures and images for: Development of a Knowledge Base for an Integrated Older Adult Care Model (SMART System) Based on an Intervention Mapping Framework: Mixed Methods Study
Source: JMIR Nurs. 2025 Aug 14;8:e59276. doi: 10.2196/59276 (PMC12352798; doi:10.2196/59276)

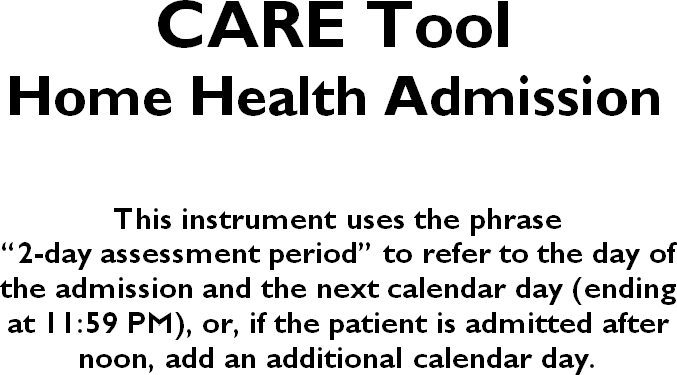


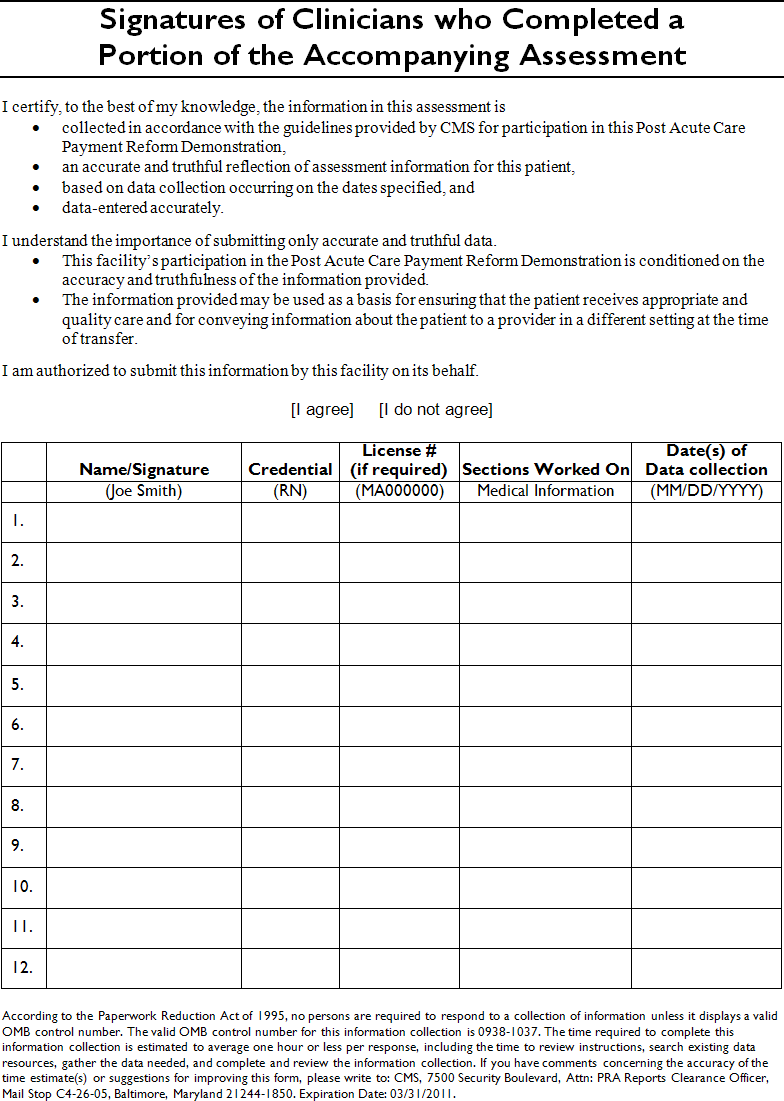


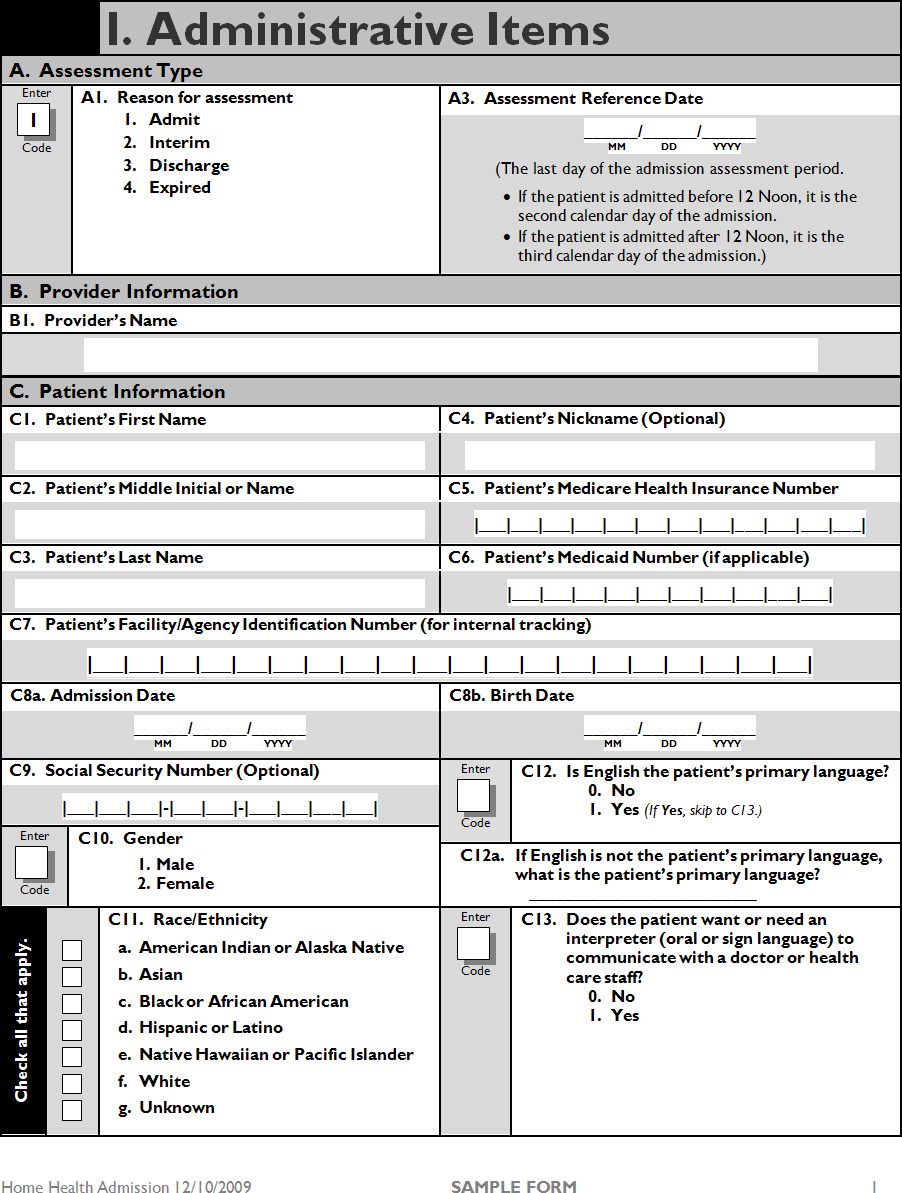


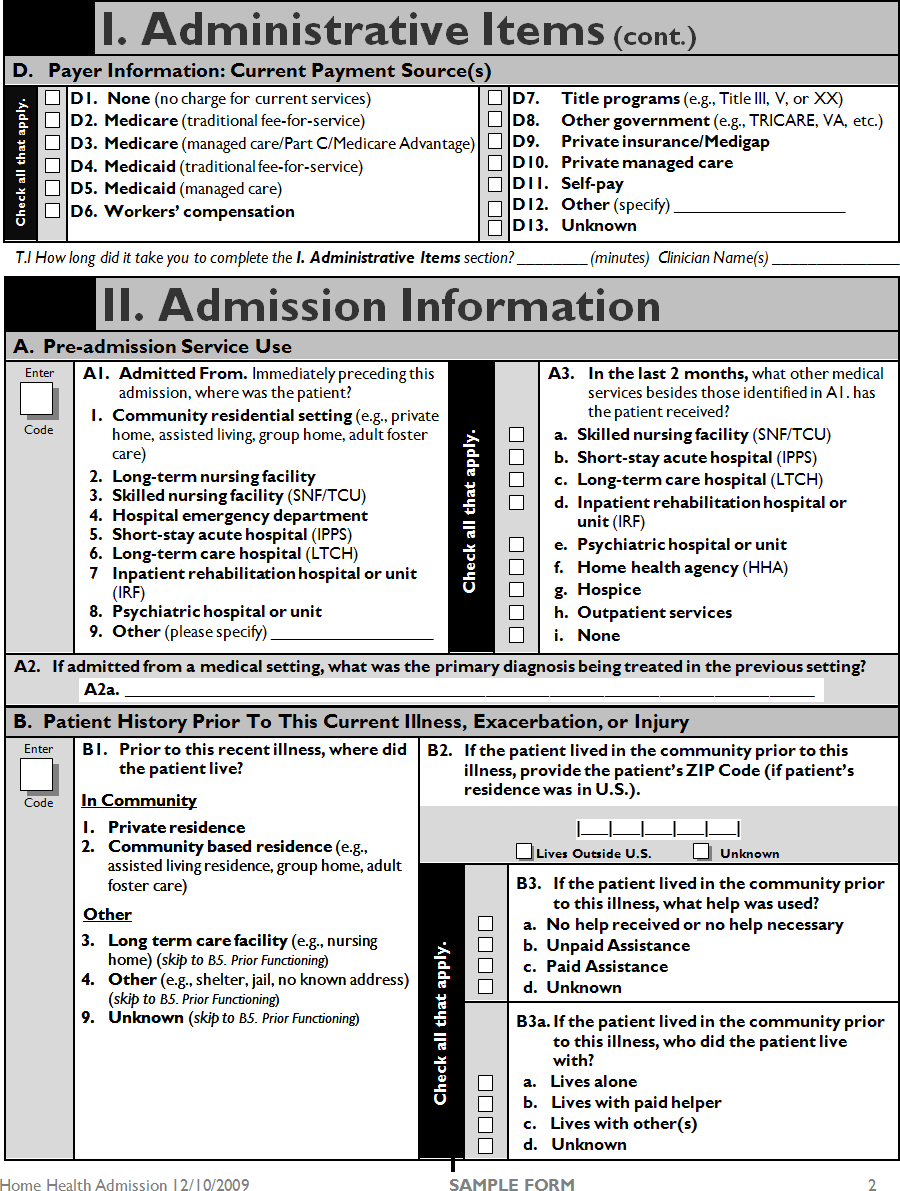


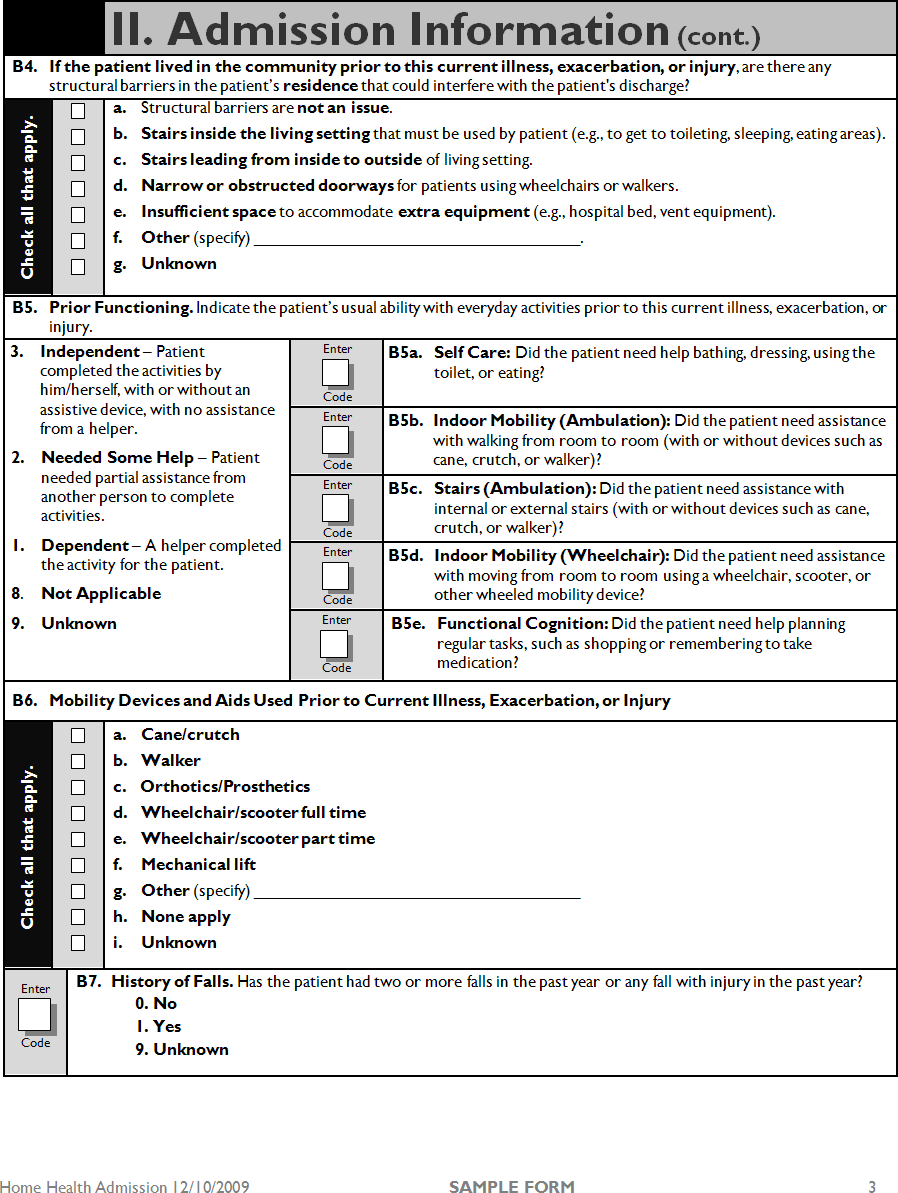


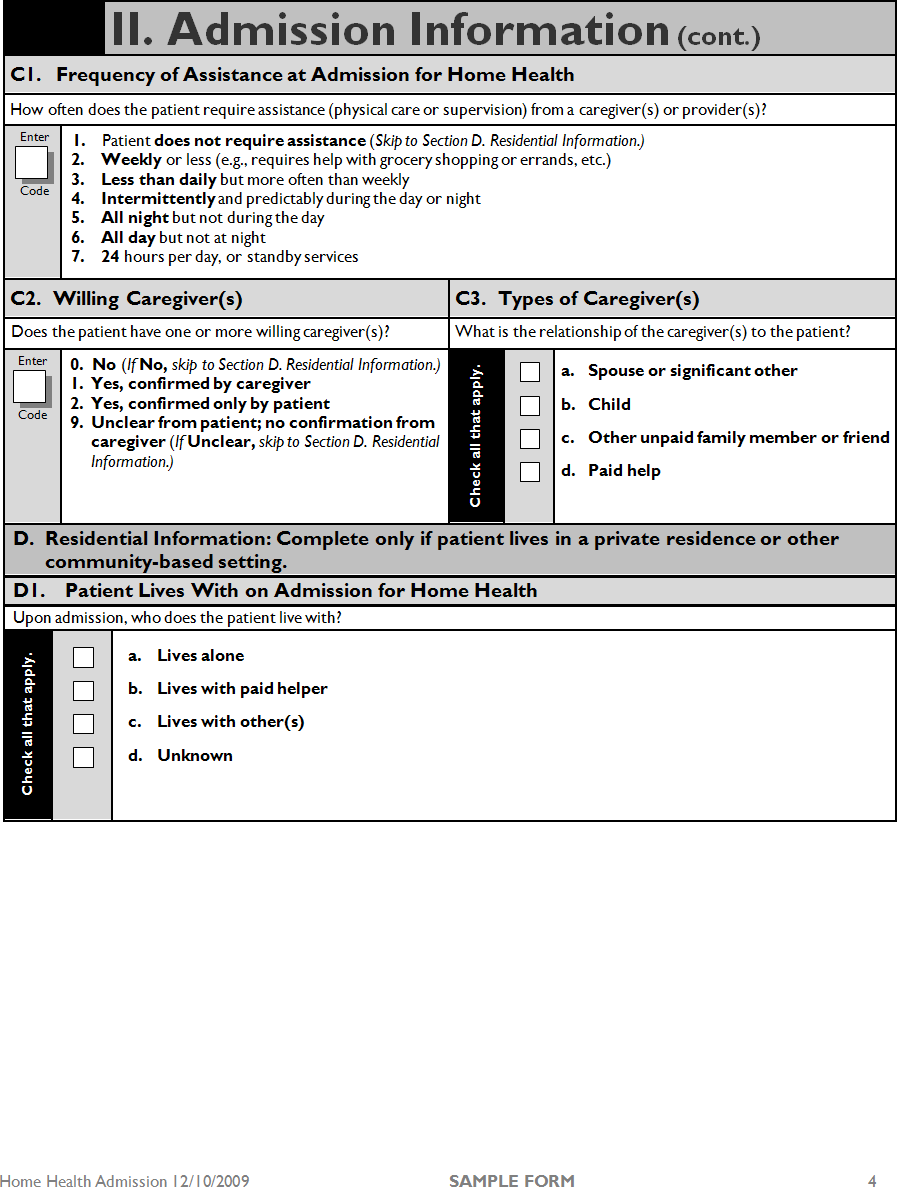


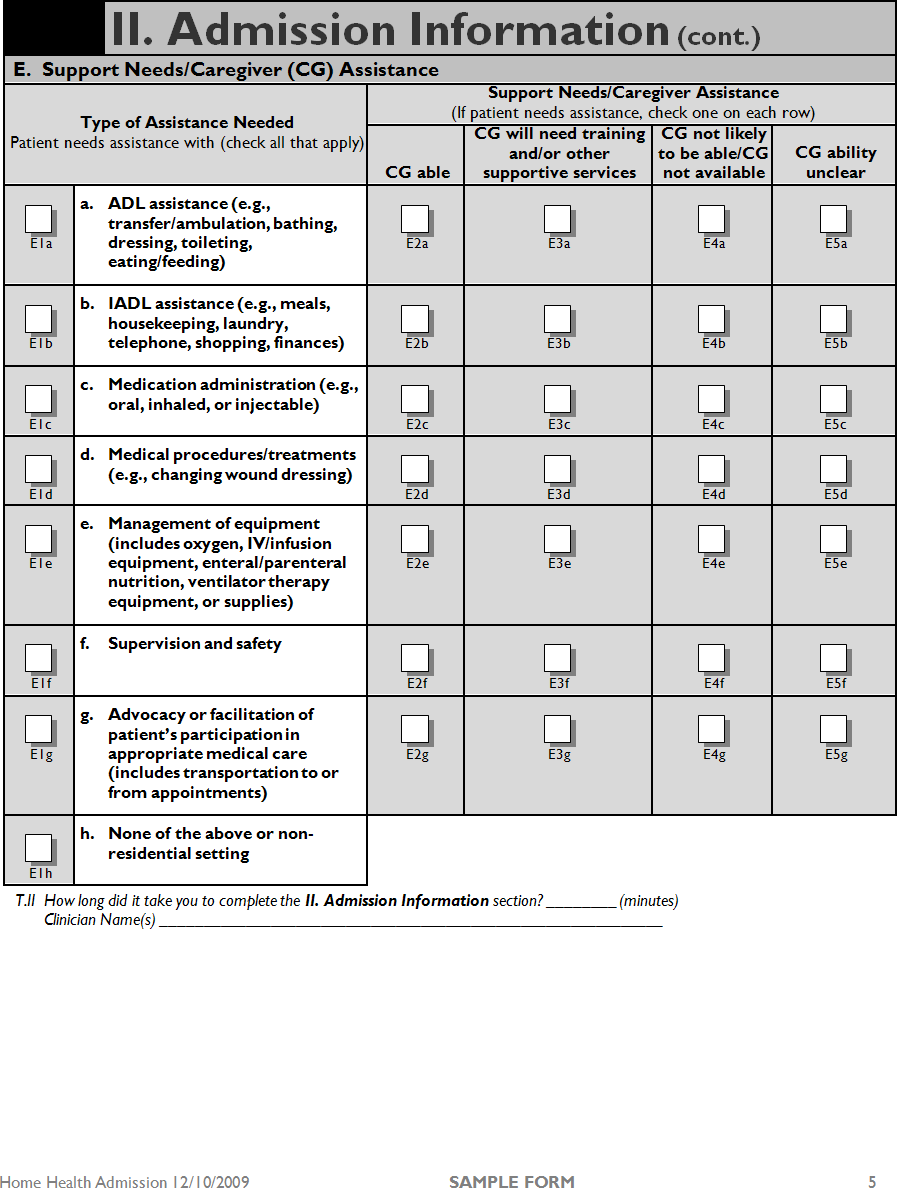


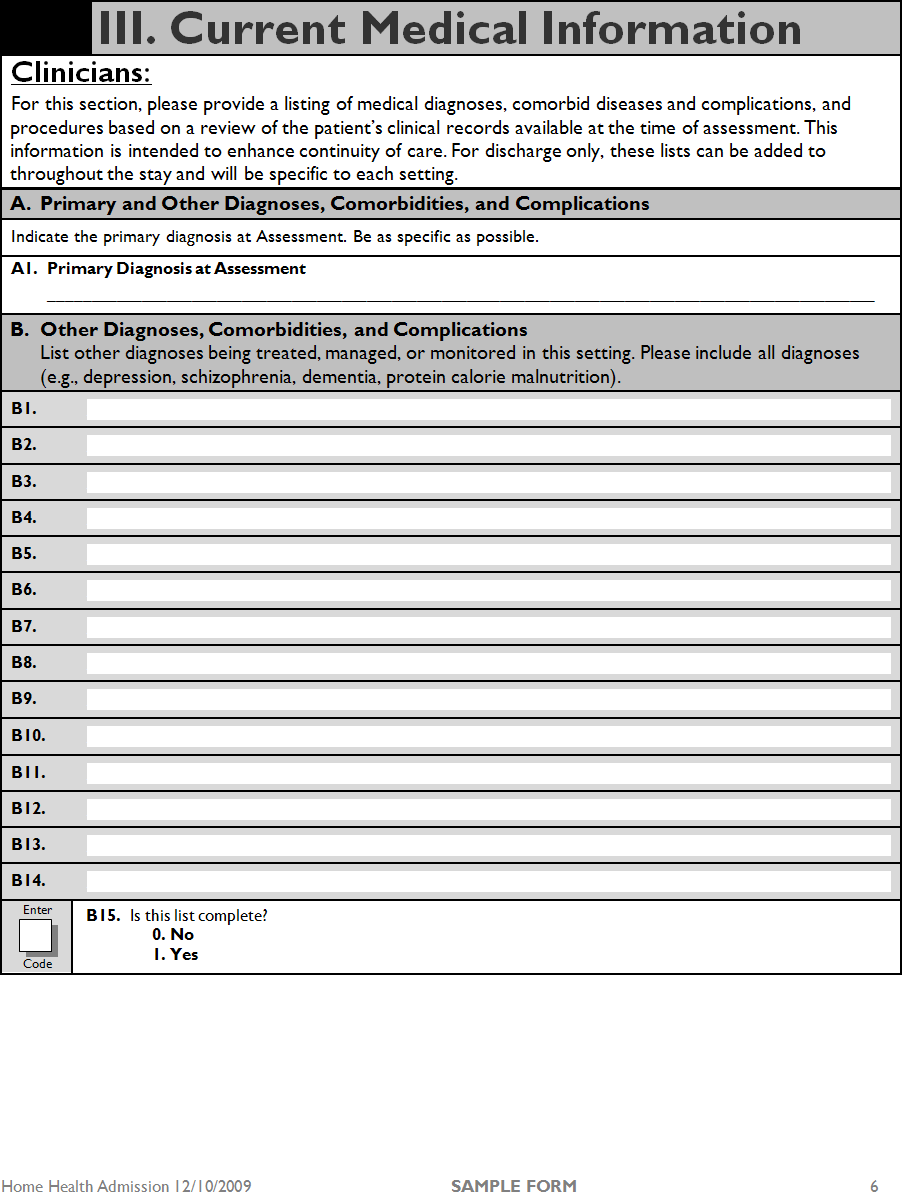


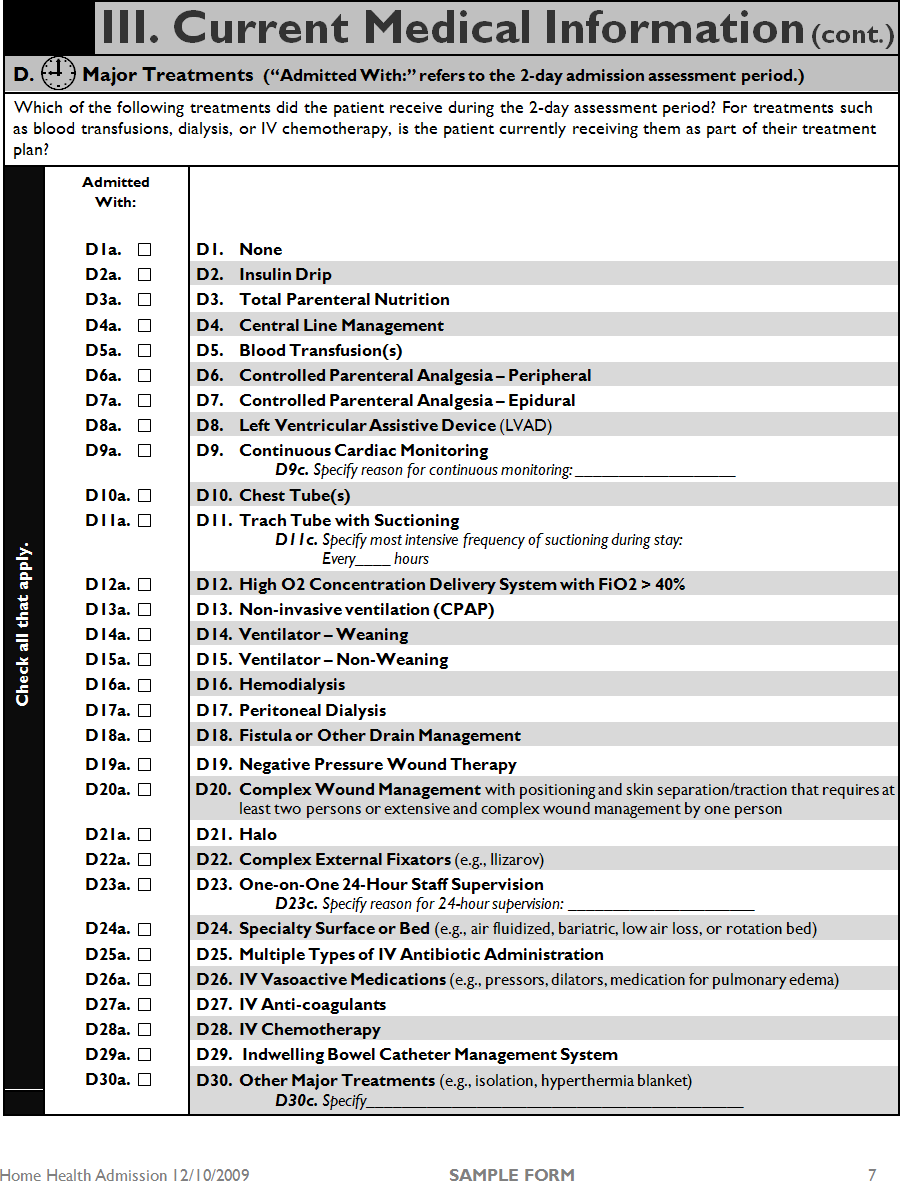


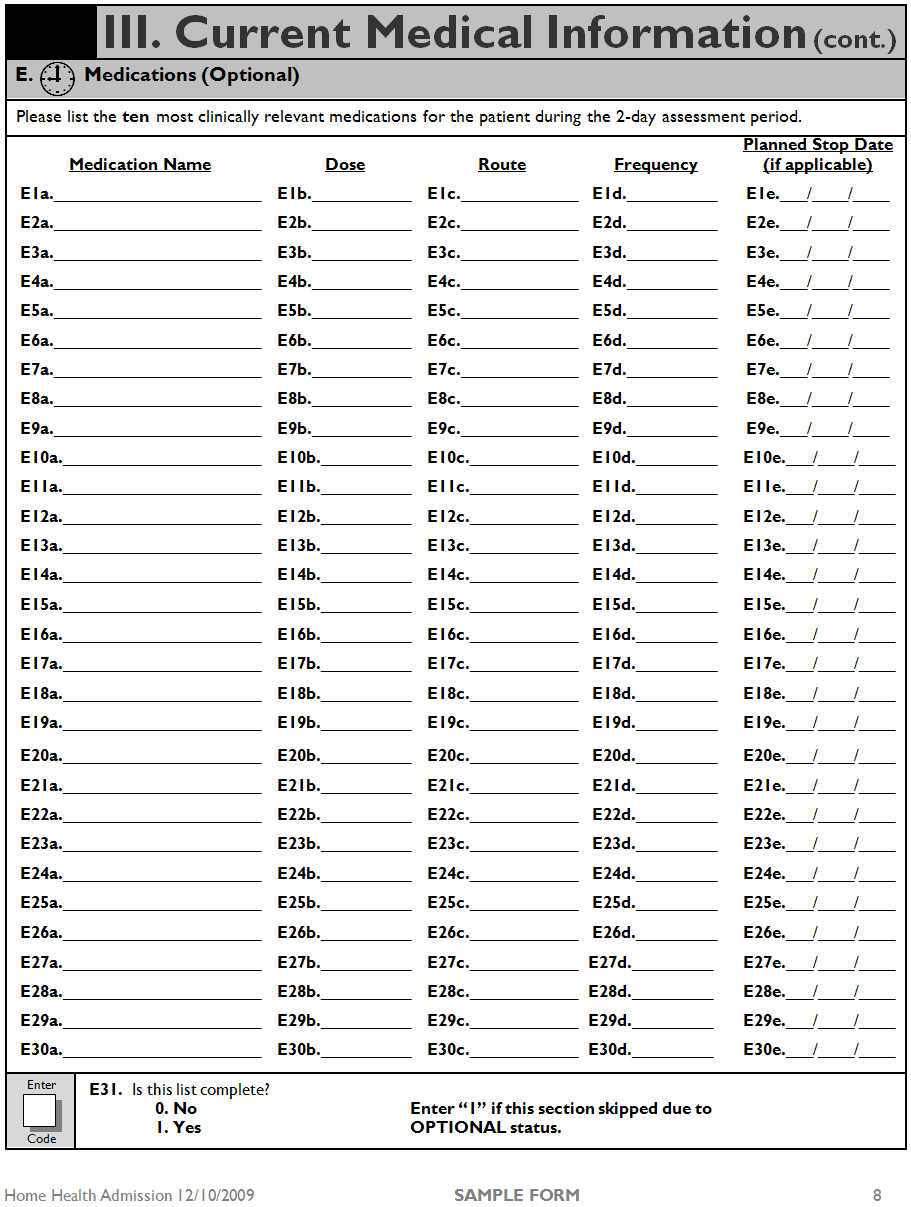


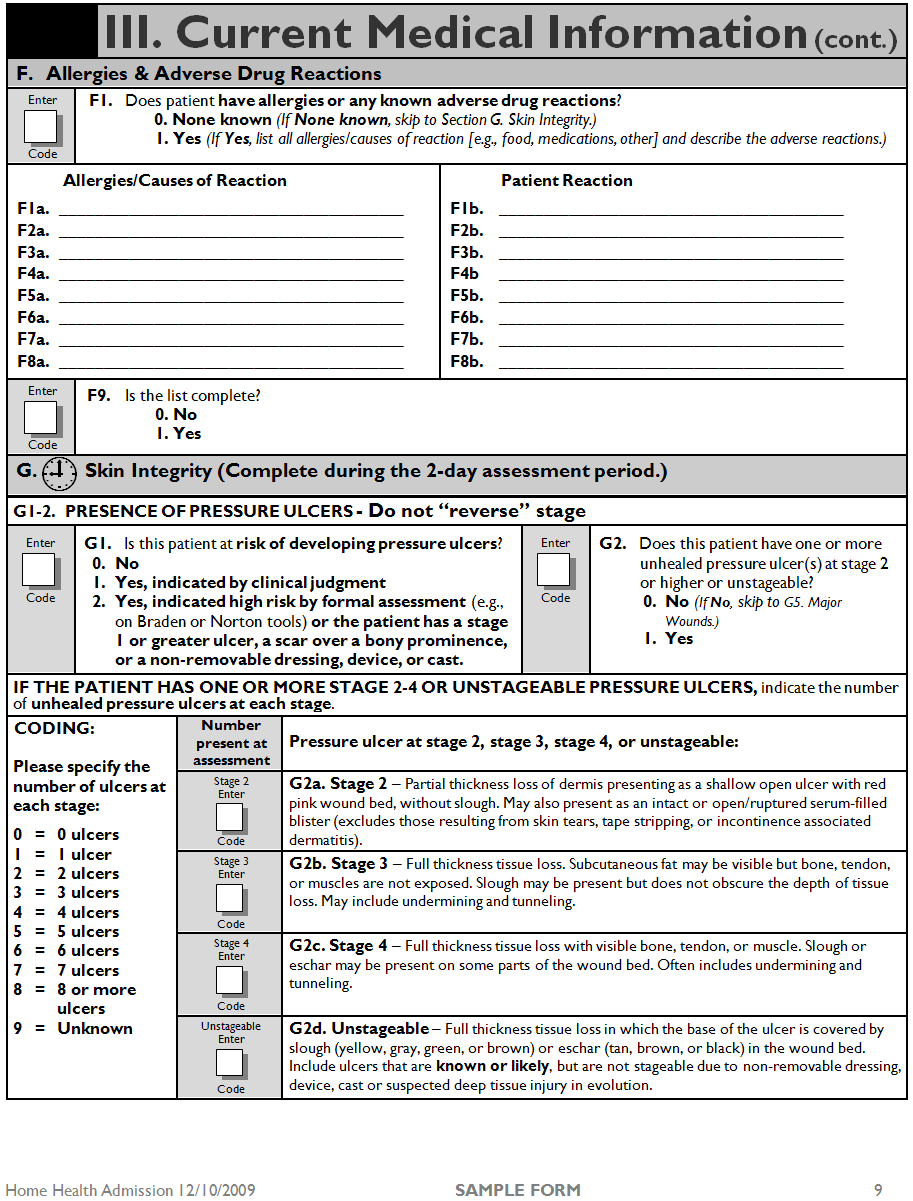


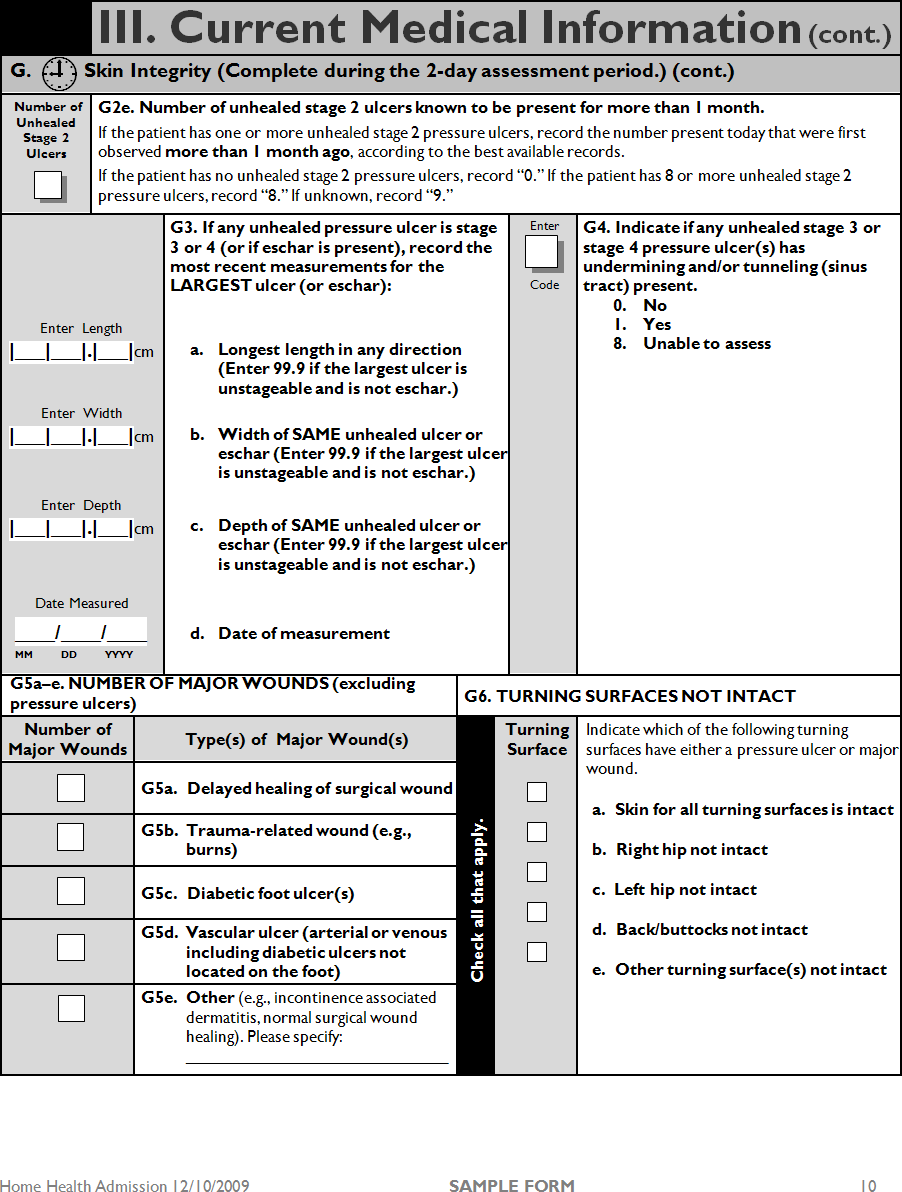


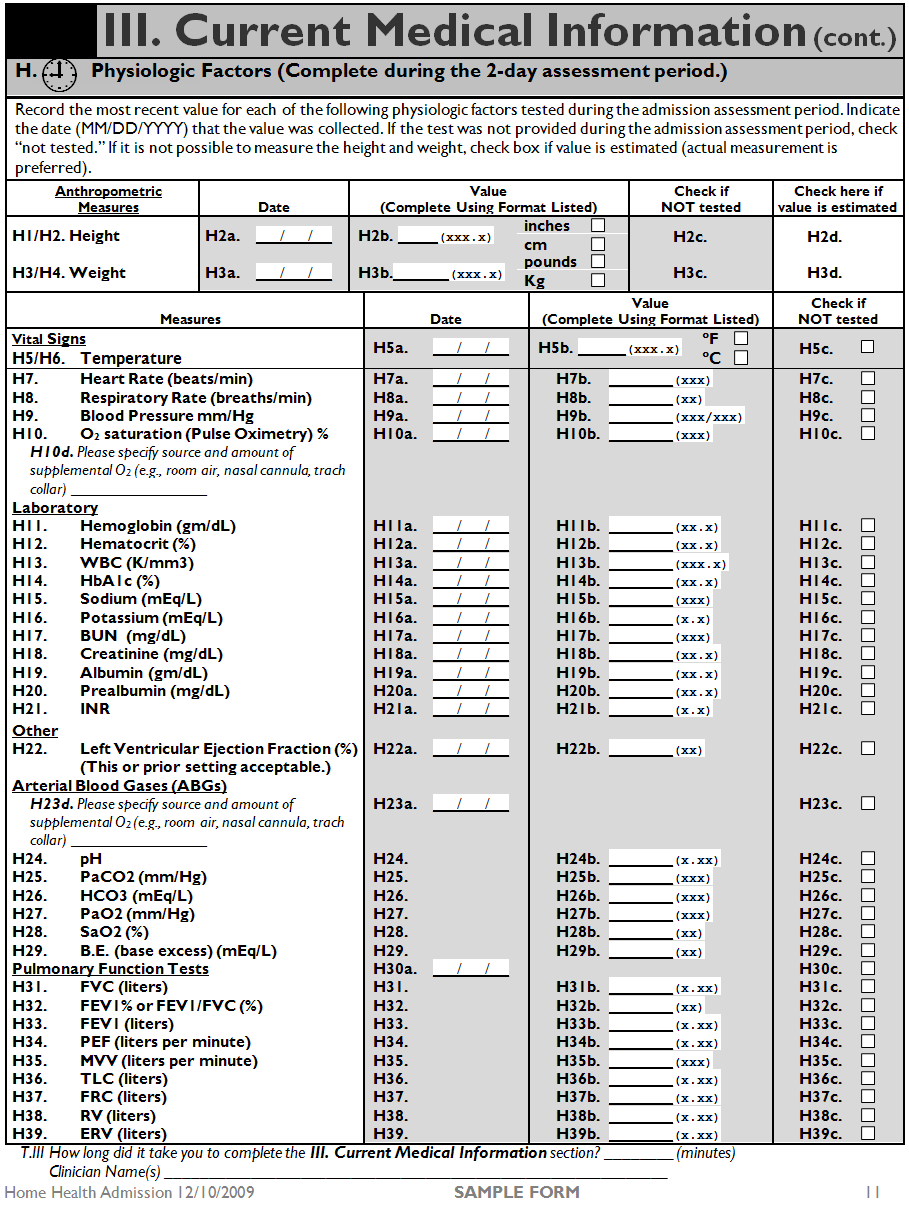


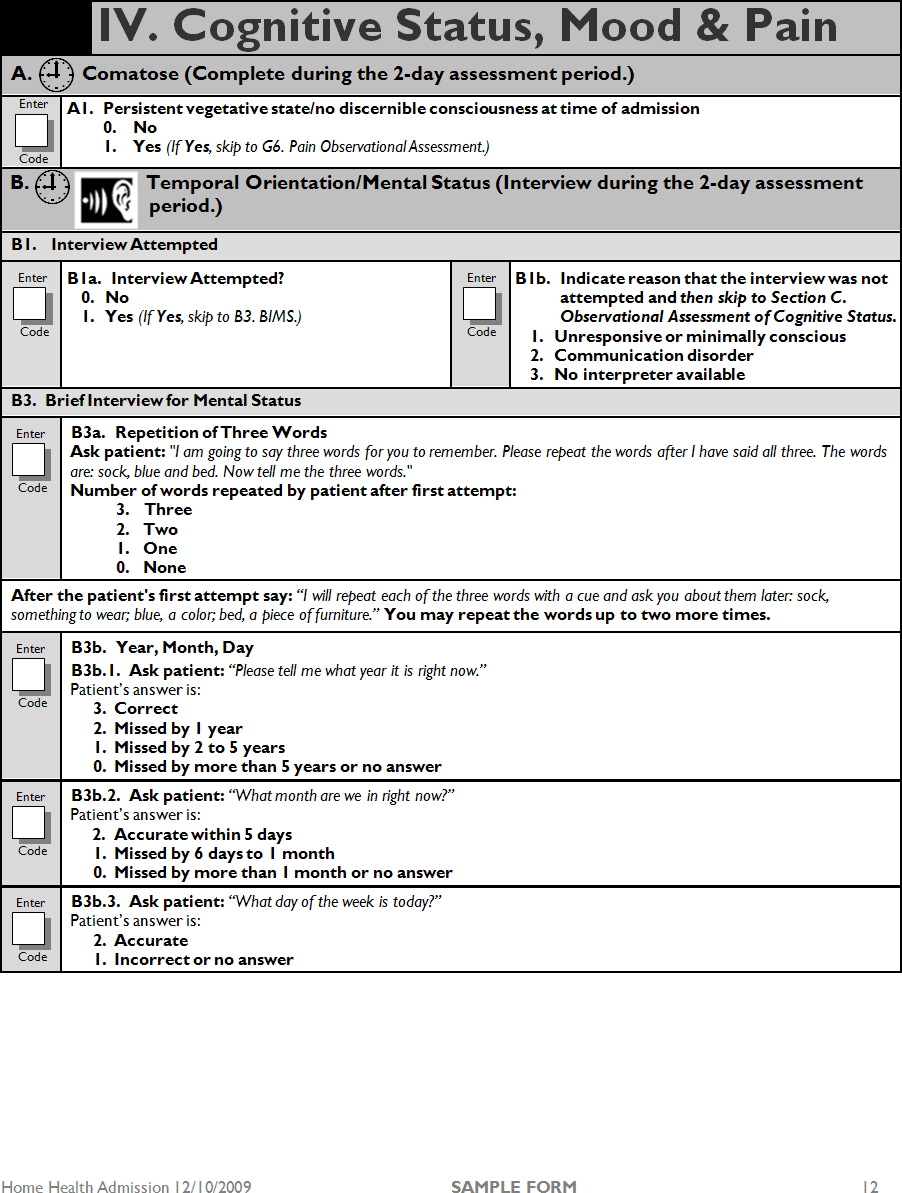


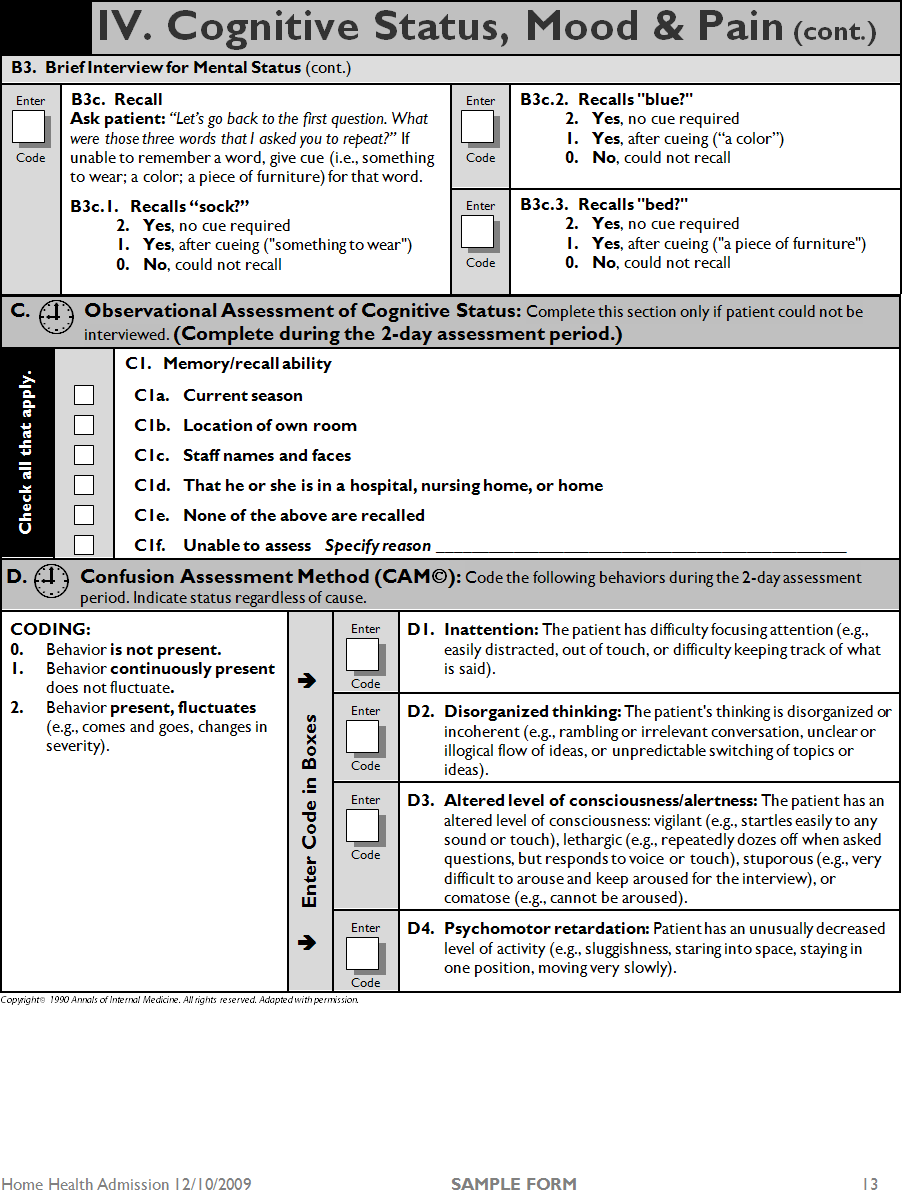


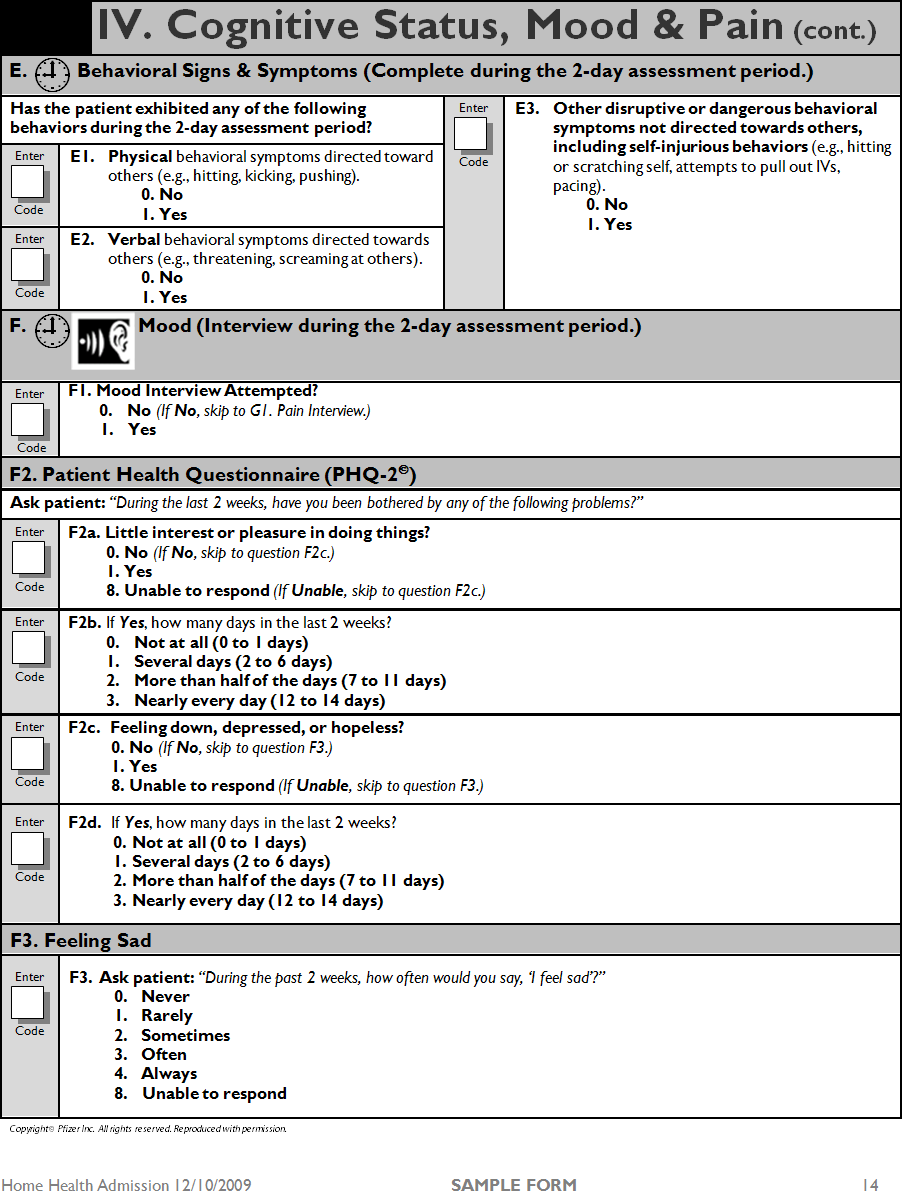


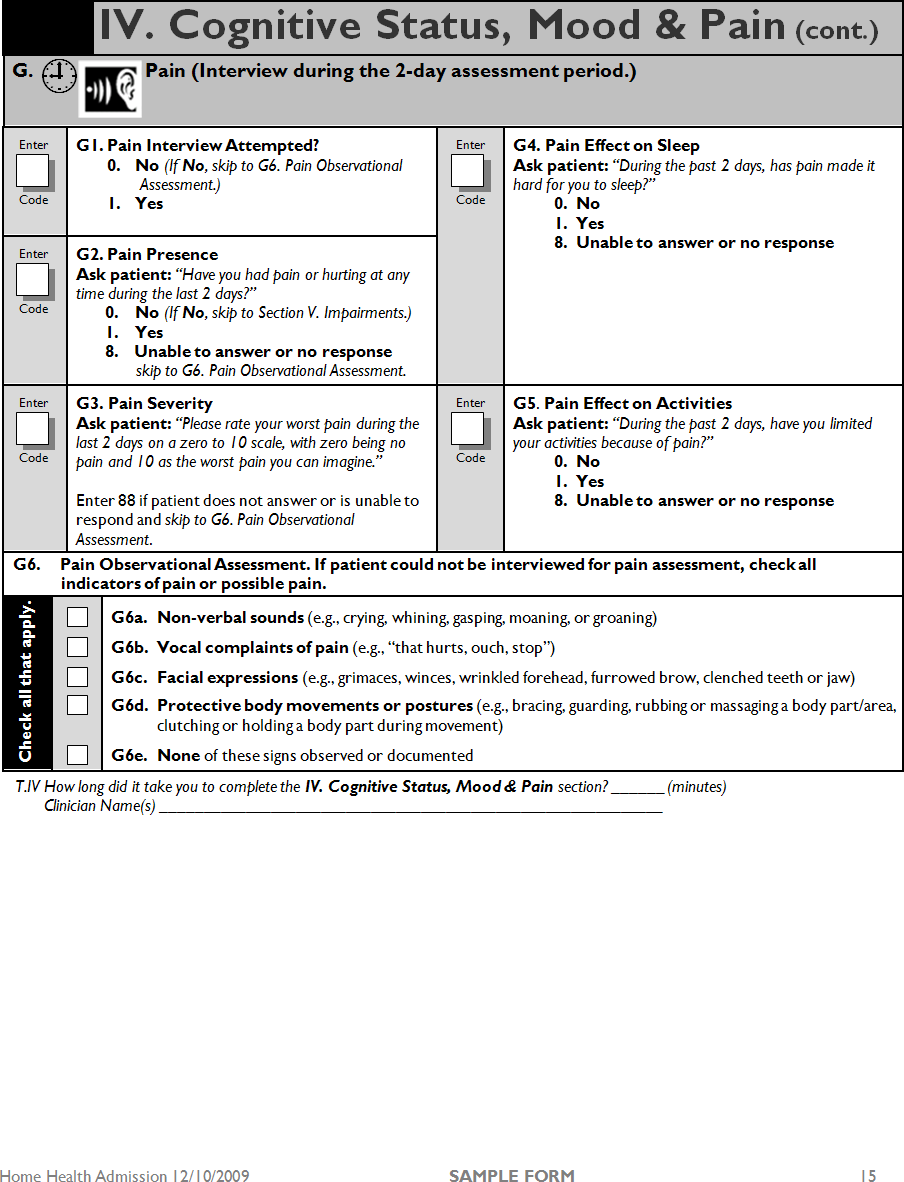


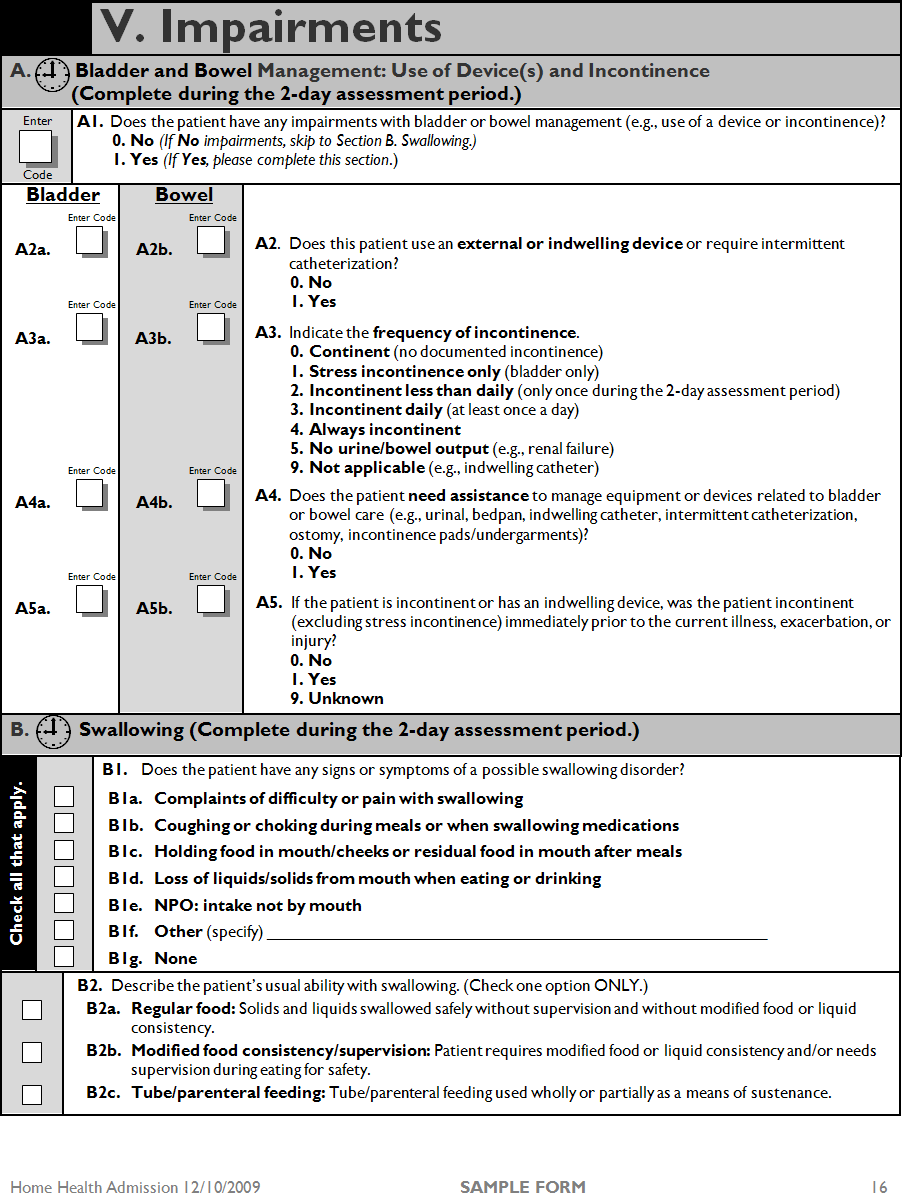


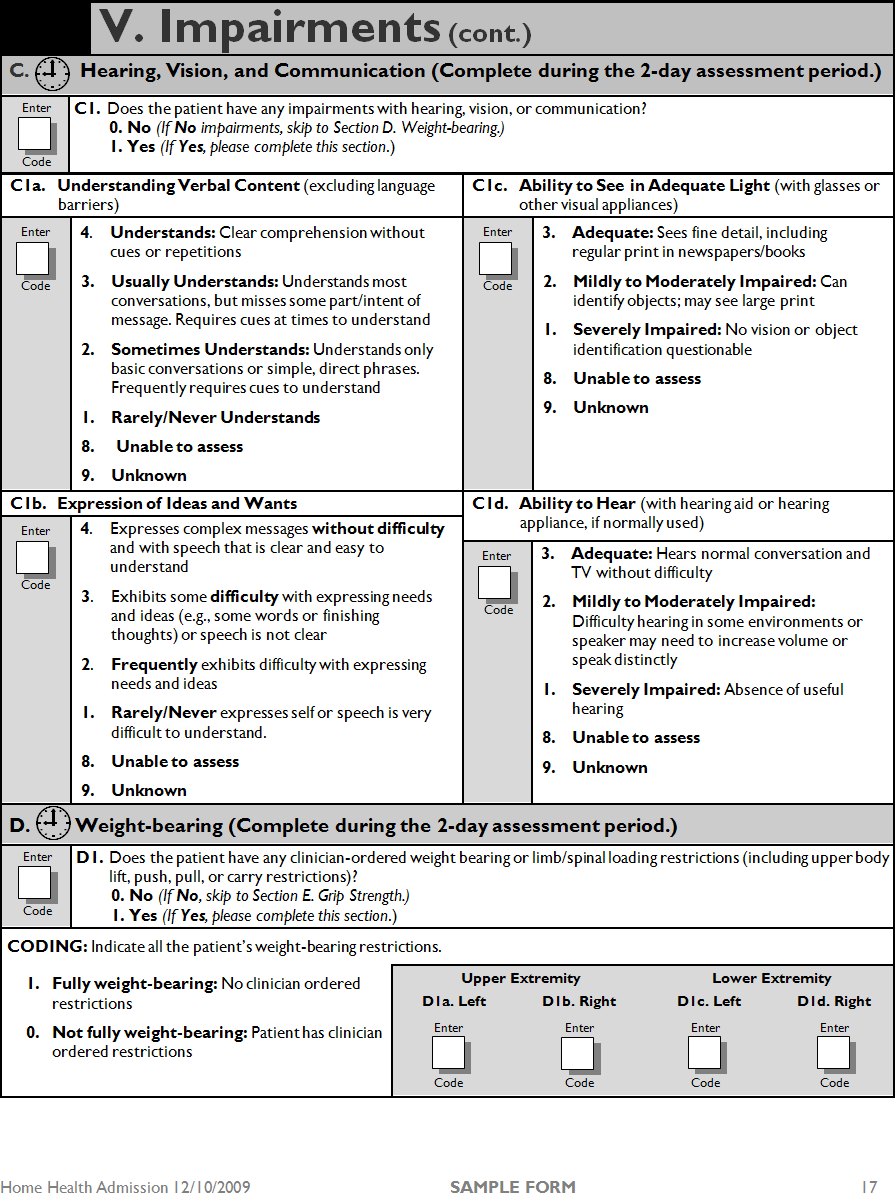


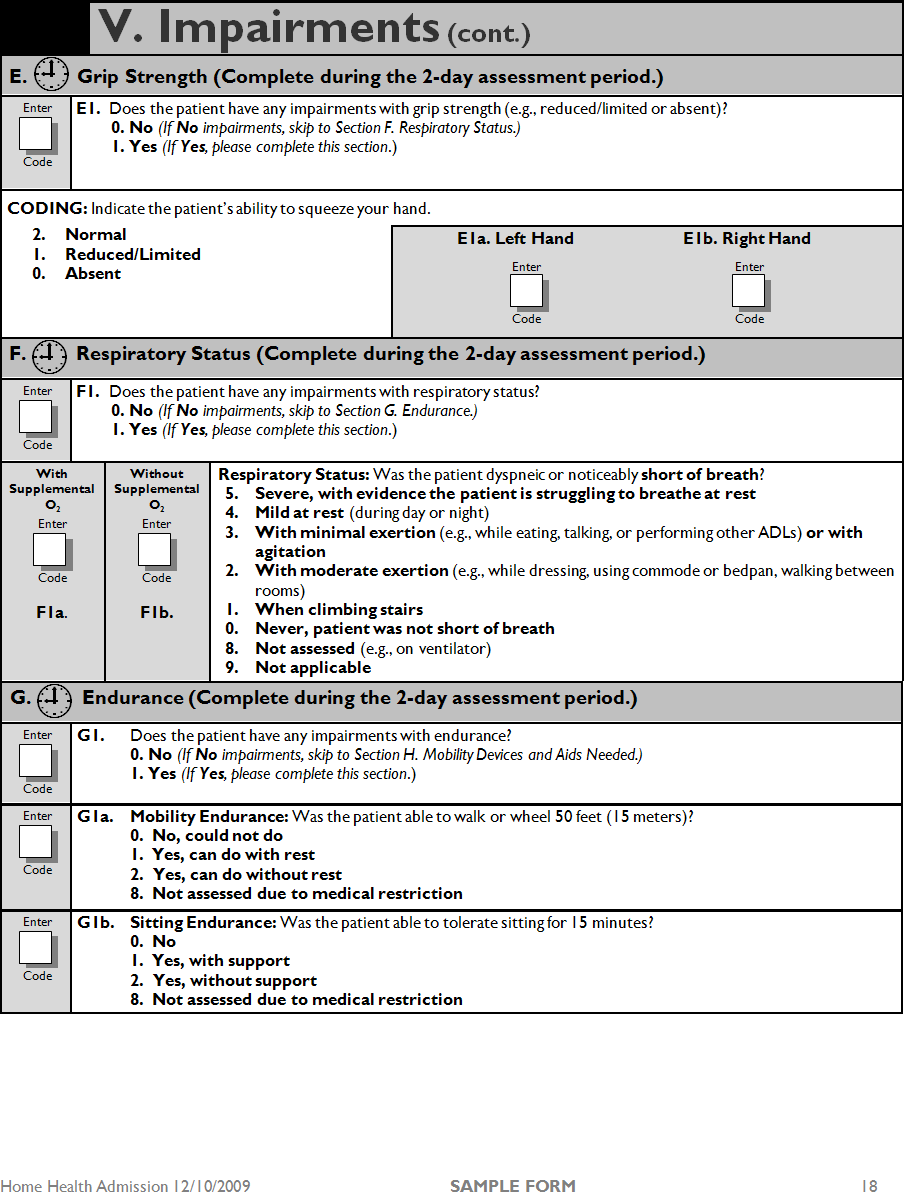


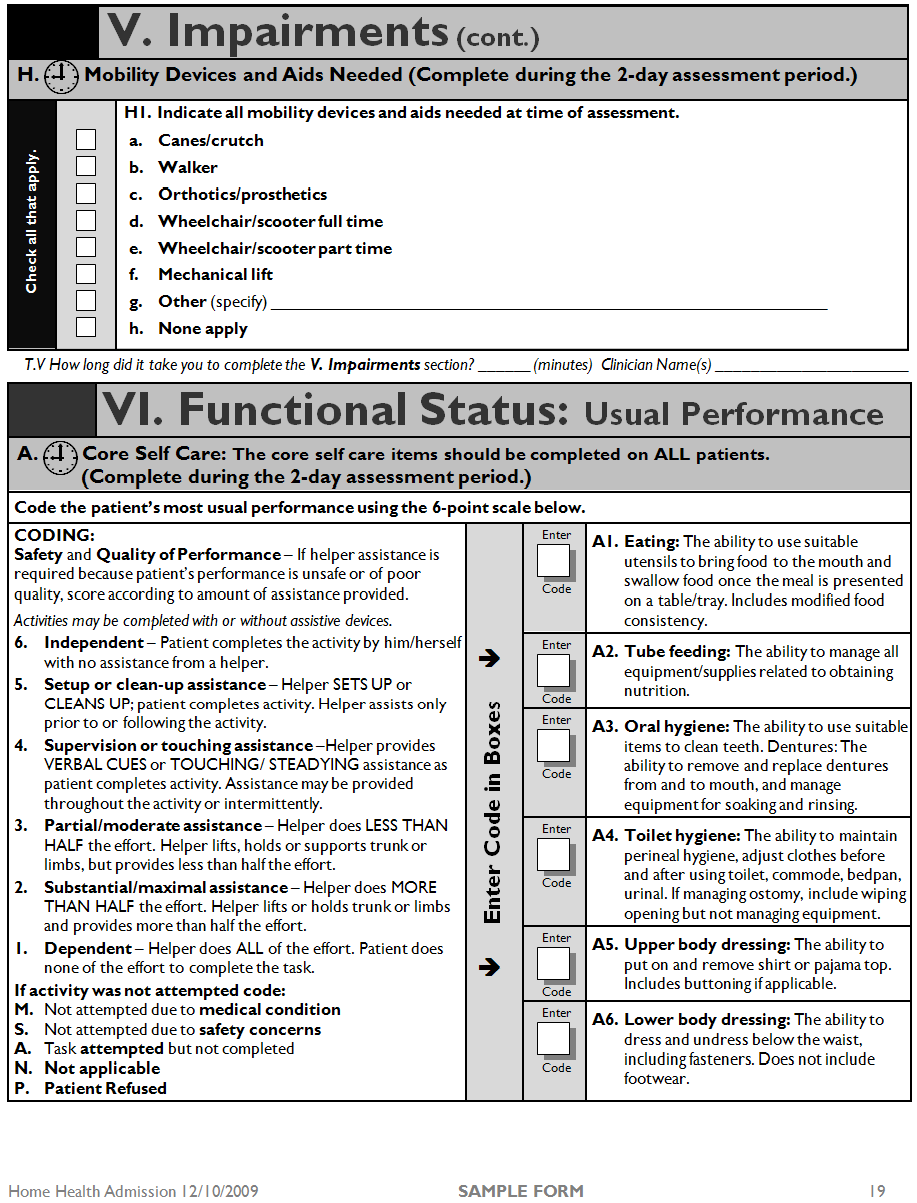


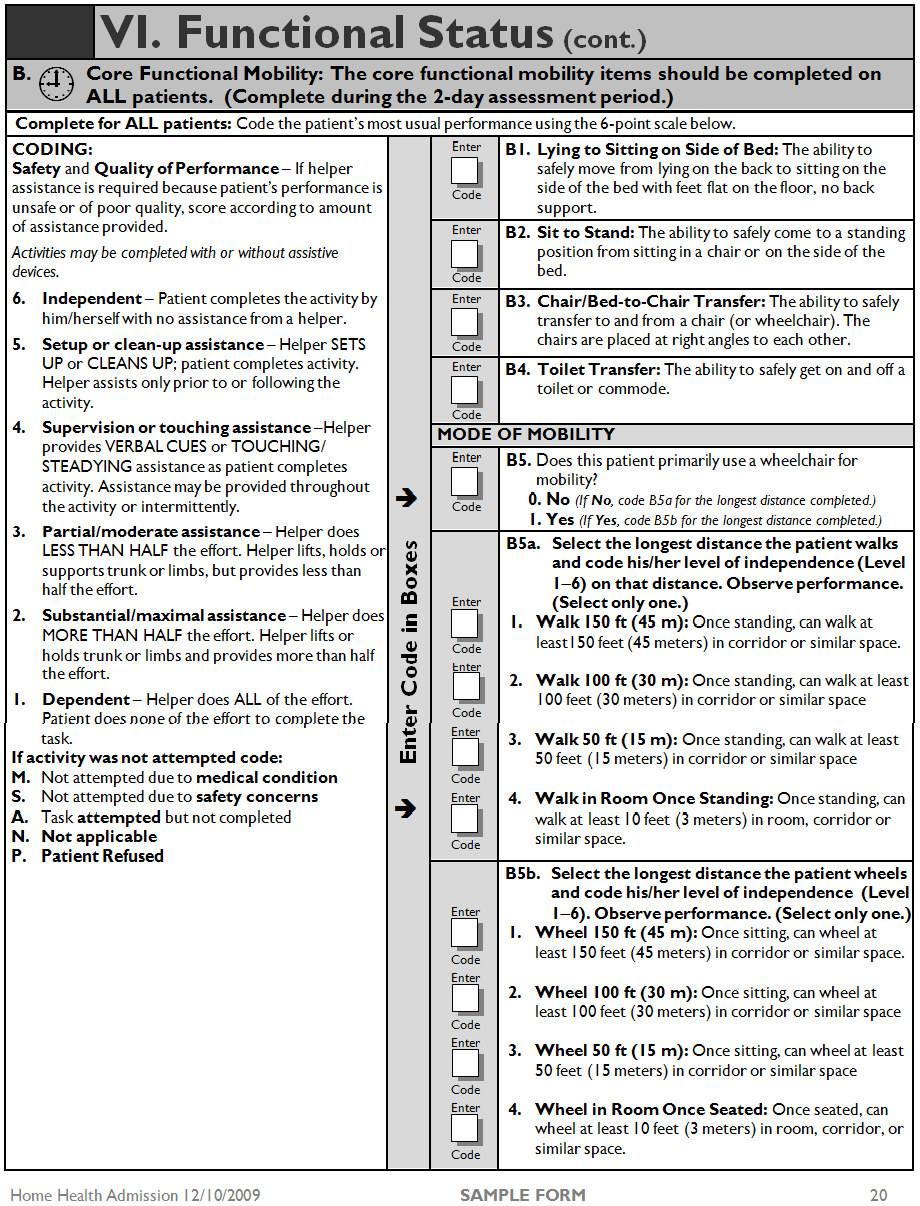


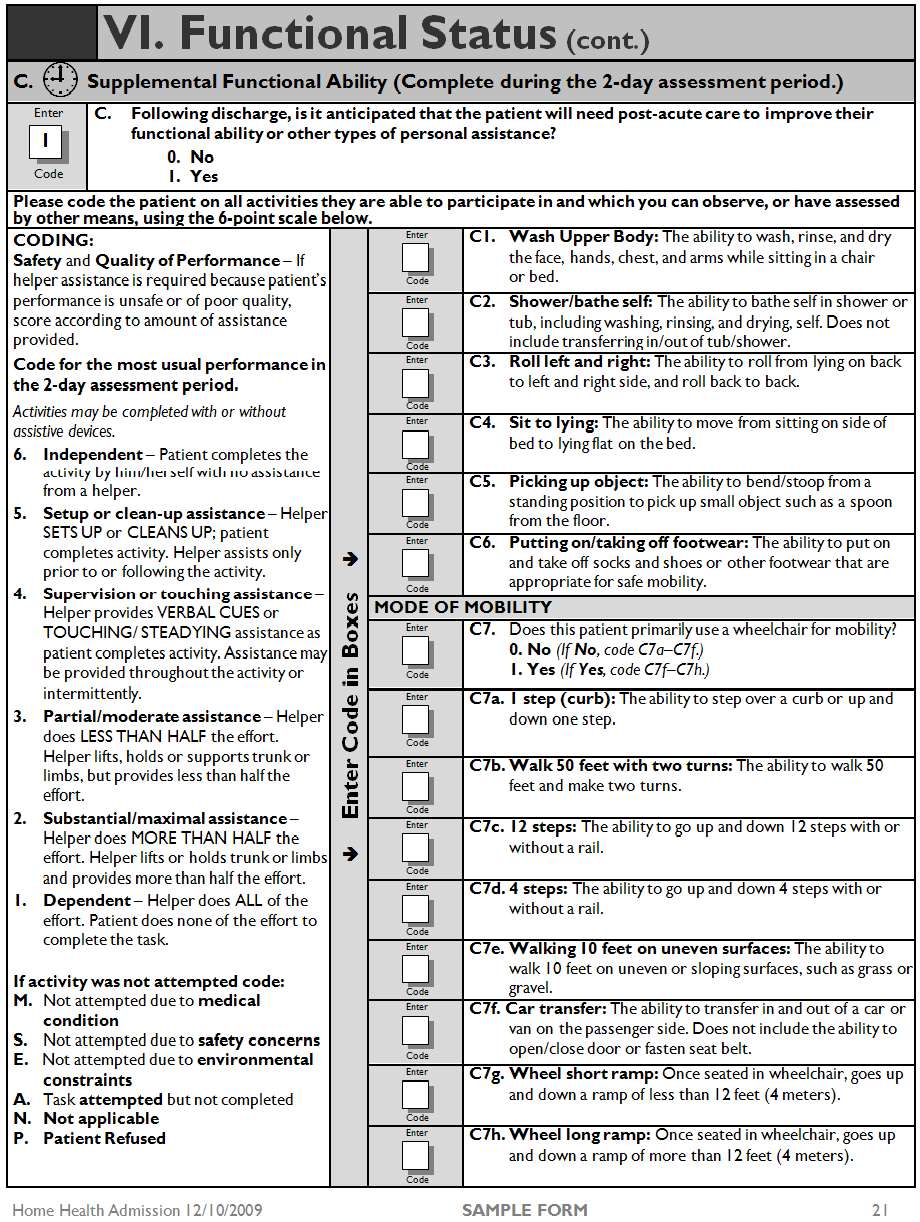


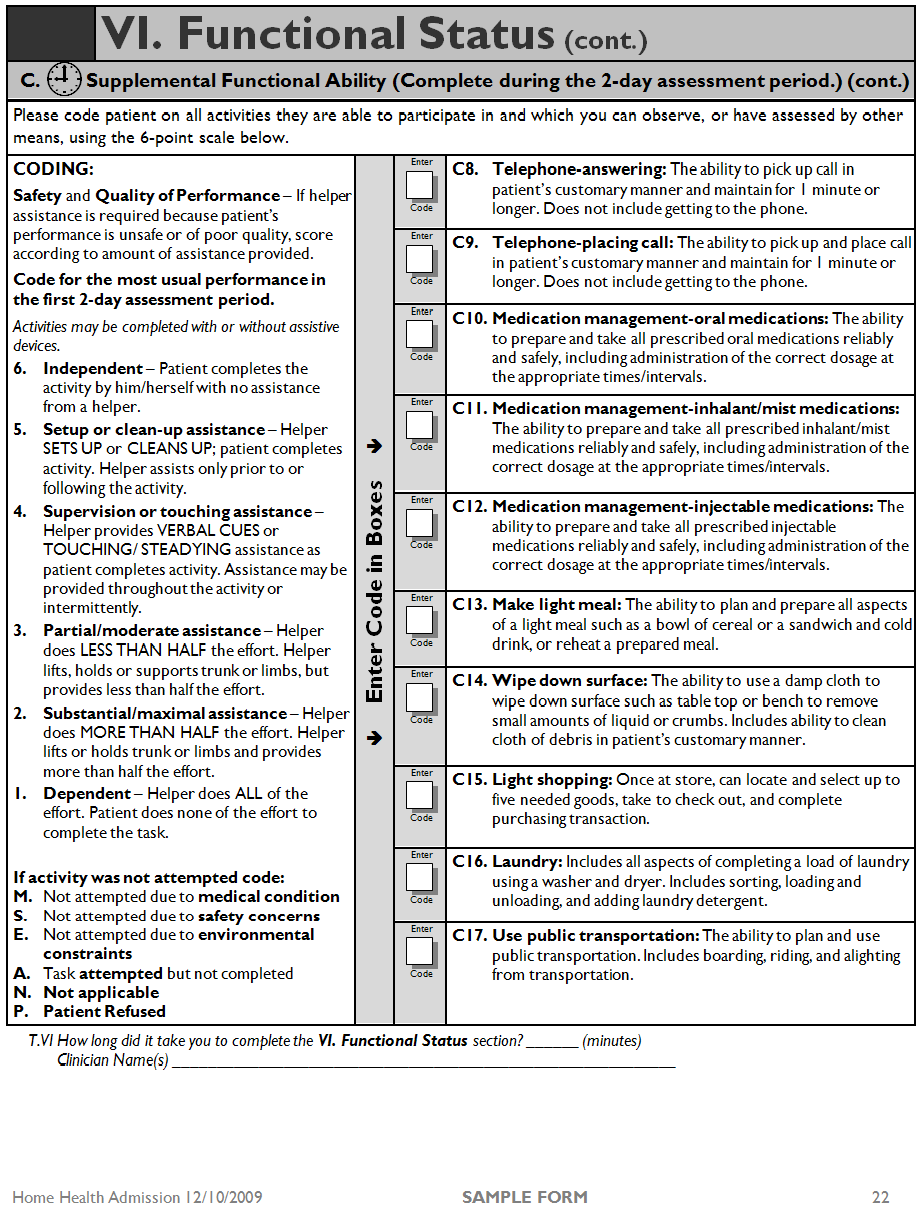


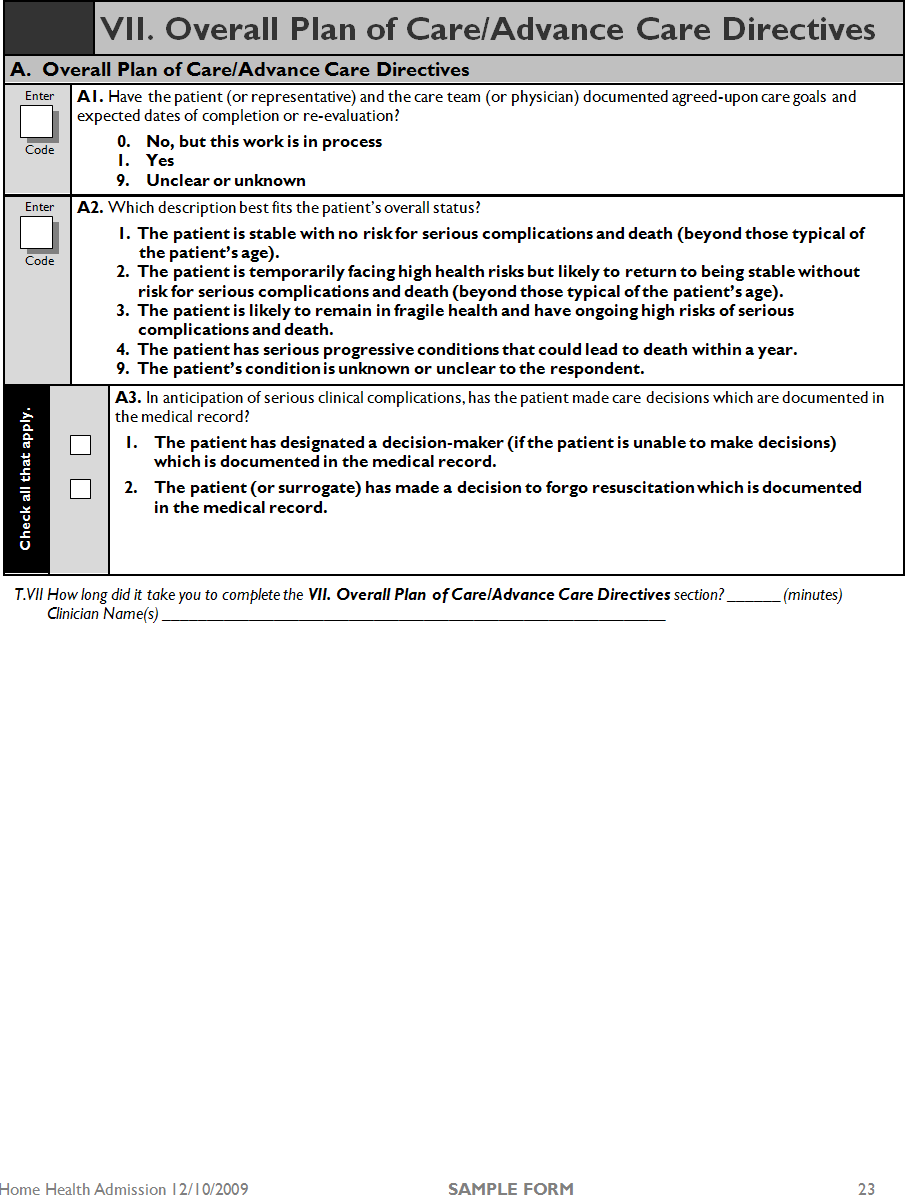


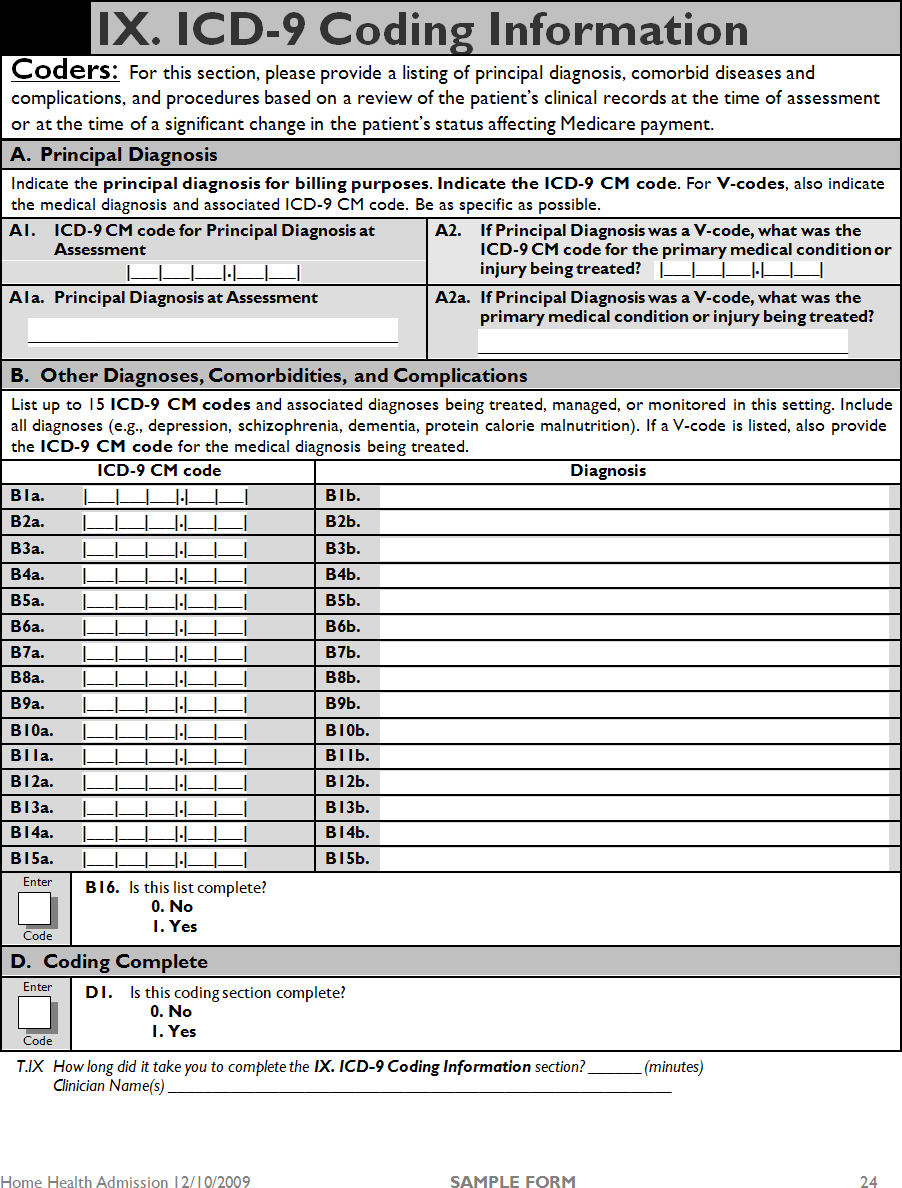


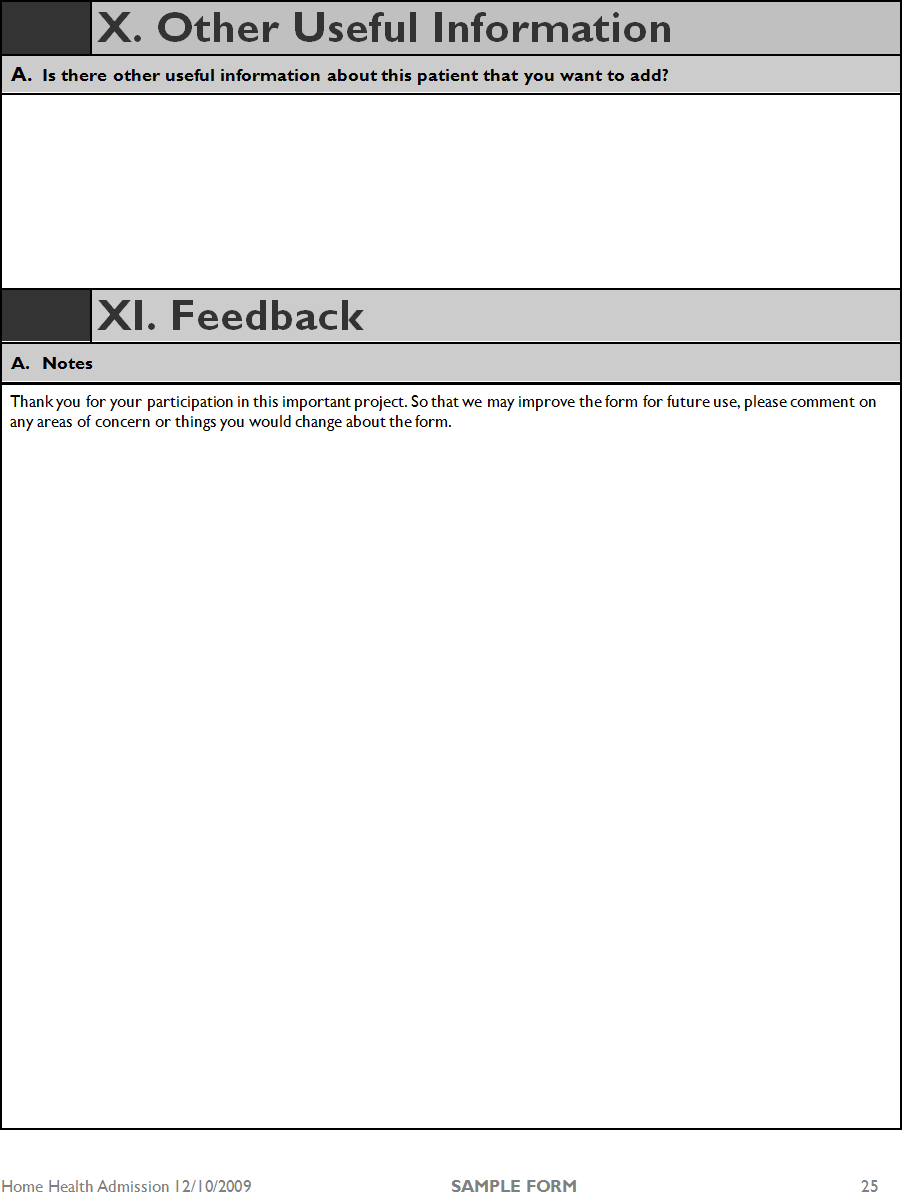

Supplement: Multimedia Appendix 1 [file nursing-v8-e59276-s001.docx]
