# Supplementary material for: Development of a Knowledge Base for an Integrated Older Adult Care Model (SMART System) Based on an Intervention Mapping Framework: Mixed Methods Study
Source: JMIR Nurs. 2025 Aug 14;8:e59276. doi: 10.2196/59276 (PMC12352798; doi:10.2196/59276)
Supplement: Multimedia Appendix 2 [file nursing-v8-e59276-s002.docx]

**File 1. Interview guide for in-depth interviews**

| 1. What are your needs for care services delivered in a home environment?  2. Why do you need care services?  3. Who is expected to provide care services for you?  4. What kind of care services do you need?  5. How often do you need care services? How long do you need care services every day?  6. What kind of contributors and barriers do you think may affect you to receive care services?  7. Have you ever thought about an App that may help provide care services and manage your daily life?  • What features do you think this App should have to make you want to use it?  • What kind of function do you want this App to be equipped with?  • What kind of presentation form that you prefer when you need to browse the information you are interested in? Text? Pictures? Cartoons? Video? Voice? And why?  • What kind of reminder do you like to have?  • During the day, when do you like to receive these messages?  8. What do you think the care services delivered via an App will bring to you and your family? |
| --- |

App, application.

**File 2. Search strategies in the PubMed database**

| **Topics** | **Search strategies** |
| --- | --- |
| Depression | (((senior) OR (older) OR (elder) OR (elderly) OR (aged)) AND (depression)) AND (interven*) |
| Fall | (((senior) OR (older) OR (elder) OR (elderly) OR (aged)) AND (fall*)) AND (interven*) |
| Social support | (((senior) OR (older) OR (elder) OR (elderly) OR (aged)) AND ((social support) OR (Social Network*))) AND (interven*) |
| Medication management | (((Polypharmacy) OR (medication compliance) OR (medication adherence) OR (drug misuse) OR (drug overuse) OR (drug overdose) OR (drug underuse) OR (drug resist*) OR (drug interaction*) OR (drug monitor*) OR (drug reaction*) OR (adverse drug*)) AND ((aged) OR (elder) OR (senior) OR (elderly) OR (older))) AND (interven*) |
| Activities of daily living | (((activities of daily living) OR (ADL)) AND ((senior) OR (aged) OR (older) OR (elder) OR (elderly))) AND (interven*) |
| Instrumental activity of daily living | (((instrumental activity of daily living) OR (instrumental ADL)) AND ((senior) OR (aged) OR (older) OR (elder) OR (elderly))) AND (interven*) |
| Emotional support | (((senior) OR (aged) OR (older) OR (elder) OR (elderly)) AND ((mental support*) OR (spiritual healing) OR (spiritual) OR (spiritual support) OR (spiritual well-being) OR (spiritual needs) OR (spiritual health) OR (spiritual care))) AND (interven*) |
| Chronic disease management | ((Chronic Disease Management) AND ((senior) OR (elderly) OR (aged) OR (older) OR (elder))) AND (interven*) |
| Disease treatment | (((aged) OR (senior) OR (elderly) OR (elder) OR (older)) AND (disease treatment)) AND (interven*) |
| Security | (((senior) OR (older) OR (elder) OR (elderly) OR (aged)) AND ((security) NOT (information security) NOT (social security))) AND (interven*) |

**File 3. Basic characteristics of interviewees enrolled in the in-depth individual interviews**

| **No.** | **Age** | **Interviewees** | **Gender** | **Cohabitants** | **Chronic diseases** | **BI** |
| --- | --- | --- | --- | --- | --- | --- |
| P1 | 88 | Older people | Male | Children | DM | 100 |
| P2 | 80 | Older people | Female | Spouse | DM, HTN, HPL, hyperostosis | 100 |
| P3 | 78 | Older people | Male | Children | DM, HTN, BPH | 90 |
| P4 | 83 | Caregiver | Female | Spouse | HTN, stroke | 35 |
| P5 | 80 | Older people | Female | Children | HTN, pelvic fracture | 90 |
| P6 | 72 | Older people/  Caregiver | Male (Female) | Children of the spouse | BC, anemia, dementia | 100 |
| P7 | 82 | Older people | Female | Children | Pneumoconiosis, premature beats | 100 |
| P8 | 81 | Older people | Male | Spouse | DM, EP | 100 |
| P9 | 84 | Caregiver | Female | Spouse | DM, HTN, CHD, BC | 15 |
| P10 | 85 | Older people/  Caregiver | Female | Spouse | DM, HTN, stroke, herpes zoster | 85 |
| P11 | 84 | Older people/  Caregiver | Female | 24-hour nanny | HTN, osteoporosis | 25 |
| P12 | 91 | Older people/  Caregiver | Female | Children | HTN, SHD, allergic asthma | 85 |
| P13 | 90 | Older people | Male | Spouse and Children | BPH | 100 |
| P14 | 62 | Older people | Male | Spouse | DM, HTN, CHD, BPH, cholelithiasis | 100 |
| P15 | 87 | Older people | Male | Spouse | BPH, pneumonia | 100 |
| P16 | 70 | Older people/  Caregiver | Female | Spouse | PD | 100 |
| P17 | 76 | Older people | Male | Spouse | HTN, stroke | 25 |
| P18 | 75 | Older people | Male | Spouse | HTN, DM, HPL, stroke | 35 |
| P19 | 66 | Older people | Female | Spouse and children | Hypomnesia | 100 |
| P20 | 67 | Caregiver | Male | Spouse and children | HTN, HPL, cirrhosis | 100 |
| P21 | 85 | Older people | Female | Spouse | DM, BC, pneumonia, cardiac insufficiency, renal insufficiency | 35 |
| P22 | 84 | Older people | Female | None | DM, HTN, right hip replacement surgery. | 80 |
| P23 | 69 | Older people/  Caregiver | Male  (Female) | Spouse | HTN, prostatitis, PD | 75 |
| P24 | 72 | Older people | Male | Spouse | HPL, stroke | 100 |
| P25 | 79 | Caregiver | Female | 24-hour nanny and children | Lung infection, heart failure, HTN, DM | 45 |
| P26 | 88 | Older people | Female | Children | Gout | 50 |

BI, Barthel index; DM, diabetes mellitus; HTN, hypertension; HPL, hyperlipidemia; BPH, benign prostatic hypertrophy; BC, breast cancer; EP, epilepsy; CHD, coronary heart disease; PD, Parkinson’s disease.

**File 4. Full details of the semi-structured in-depth interview results**

***Older people’s needs for daily life care***

**Diet care:** Older people’s needs for diet care are generally simple with their family members or caregivers as the main care providers and the community senior table as a supplementary care provider. Eleven older individuals stated their needs for diet care:

*I’m having trouble cooking for myself now. When my children are at home, they go shopping and cook for me. But when they go to work, I have to eat at the community senior dining table.* [P13, Male, 90 years old]

**Mobility care:** Mobility care refers to a certain degree of support in walking, moving, turning over, and other actions for older people to avoid the occurrence of adverse events such as falls, falling out of bed, and sudden onset of diseases. Six older individuals shared their needs for mobility care:

*I’m really afraid of falling down when I go out alone. Whenever I go out, I want to have someone with me.* [P5, Female, 80 years old; P19, Female, 66 years old]

*I need help getting in and out of bed and even turning over in bed. Actually, I feel that I can’t do anything without* *assistance from others.* [P21, Female, 85 years old]

**Sleep care:** Poor sleep quality is a problem commonly encountered by older adults. Sleep care refers to providing some measures to improve older people’s sleep quality, including appropriate medication use, guidance on the sleep environment, traditional Chinese medicine modalities, etc. Two older individuals revealed their needs for sleep care:

*My sleep quality is quite poor, and even the slightest noise at night prevents me from falling asleep. I typically resort to taking two sleeping pills when sleep eludes me, but recently, this method has become less effective. Therefore, finding effective strategies to improve my sleep quality is crucial for me.* [P2, Female, 80 years old]

**Security care:** Security care refers to providing guidance on older-oriented renovations for older people’s living places and daily necessities to avoid accidents and ensure their basic security. Two older adults shared their needs for security care:

*I am earnestly seeking guidance regarding my home environment, furniture, and daily use items. Given my condition of diabetic feet, specific recommendations on choosing appropriate shoes and socks would be particularly beneficial for me.* [P14, Male, 62 years old]

**Personal Hygiene and Grooming:** Many older people struggle with maintaining personal hygiene due to limited mobility or cognitive decline. Assistance with bathing, oral care, and grooming is crucial to promote dignity and prevent infections.

*I am unable to bathe myself, and I rely on my daughter to help me with everything from washing to brushing my teeth.* [P25, Female, 79 years old]

***Older people’s needs for healthcare***

**Expanding health knowledge:** Health knowledge here refers to information crucial for disease prevention, disease management, and prevention of related complications. Five older individuals expressed their viewpoints about their need for health knowledge:

*Following my diagnosis of stroke and hypertension, I found myself uncertain about how to maintain health. For instance, while I am aware of the need for a healthy diet to manage my blood pressure, I am unclear about which foods I should consume more of and which ones to reduce.* [P4, Male, 41 years old]

**Available consulting services:** Consulting services are defined here as channels through which older individuals can seek advice from professional doctors or nurses regarding their symptoms and concerns. Five older people revealed their needs for the availability of these services:

*Approximately a month ago, I noticed a marked decrease in the frequency of my bowel movements, though there were no instances of shapeless stools or hematochezia. This observation led to my concern about the possibility of colorectal cancer. Had a doctor been able to assure me at that time that cancer was not present, it would have greatly alleviated my anxiety.* [P8, Male, 81 years old]

**First aid:** First aid for older individuals, particularly those living alone, involves ensuring access to necessary emergency equipment and services. Two older individuals specifically expressed their need for such emergency rescue facilities:

*We find it quite challenging to make phone calls for assistance during emergencies or unexpected incidents. Therefore, the widespread implementation of an emergency bell system would be extremely beneficial, in my opinion.* [P12, Female, 91years old; P16, Female,70 years old]

**Rehabilitation training guidance:** Rehabilitation training guidance is urgently needed to enable older people’s ability to live independently and maintain their highest possible quality of life. Four older individuals expressed their needs for rehabilitation training guidance:

*Ever since my leg was broken, my ability to walk independently has been compromised. I am keenly interested in receiving professional rehabilitation guidance to aid in the recovery and restoration of my walking ability.*[P7, Female, 82 years old]

*Following surgery for Parkinson’s disease, my hands constantly tremble, to the extent that I struggle to hold chopsticks. My doctor advised that loudly reading newspapers and engaging in rehabilitation exercises could facilitate my recovery. However, I often find myself uncertain about whether I am performing these activities correctly.* [P16, Female, 70 years old]

**Medication Management:** Older people often experience polypharmacy and may forget to take their medication or take it incorrectly. Three older individuals expressed their needs for medication reminders:

*I have too many pills to take every day. Sometimes I forget whether I’ve taken them or not.* [P19, Female, 66 years old]

*Sometimes I feel like the medicine doesn’t work at all. So I just take a few more tablets, hoping it’ll help faster. But I’m not sure if that’s the right thing to do.* [P23, Male, 69 years old]

*There are days when I think taking the medicine is pointless, so I just skip it altogether. I don’t know if that’s okay or if I should stick to what the doctor said.* [P24, Male, 72 years old]

***Older people’s needs for external support***

**Psychological support:** Psychological support holds critical importance for the elderly, as it substantially influences their physical and mental well-being, along with their ability to adapt socially. Two older individuals specifically articulated their need for such psychological support:

*The diagnosis of a chronic disease can be overwhelming, often giving rise to the mistaken belief that life will inevitably become a series of challenges. This is compounded by the pervasive notion that all diseases are incurable. Such circumstances can also lead to a sense of isolation, as there is a perception that the emotional impact of the diagnosis is not fully understood by others.* [P10, Female, 85 years old]

*I have no one to keep me company and bring me happiness, except for the nanny at home.* [P11, Female, 84 years old]

**Wide social network:** Family members and caregivers play a pivotal role in offering physiological and psychological support to older adults. Additionally, neighbors and friends can contribute significantly to their well-being by providing companionship. Consequently, broadening the social networks of older adults holds substantial value. Four older individuals expressed their concerns regarding the extent of their social connections:

*My interactions with neighbors are limited, and I occasionally converse with my retired colleagues. However, after their passing, I’ve found myself without anyone to talk to, leaving me feeling deeply lonely.* [P16, Female, 70 years old; P17, Male, 76 years old]

**Available healthcare services at community:** The gradual decline in physiological functions commonly experienced by older individuals often necessitates their reliance on healthcare services within their community. Four older persons expressed a desire for accessible community healthcare services:

*It would be beneficial if simpler medical treatments were available within the community. For instance, the infusion services currently offered locally are a convenient choice for hospital visits, saving us considerable time and energy.* [P10, Female, 85 years old]

*To better address minor ailments within the community, community health centers need to employ more experienced professional doctors and nurses. This approach would enable more effective treatment of these conditions locally.* [P3, Female, 78 years old; P16, Female, 70 years old]

***Older people’s needs for social participation and self-development***

**Social participation:** Older individuals require a range of social and recreational activities to fulfill their needs for social interaction, learning new knowledge and skills, and achieving self-fulfillment. Interviewee #21 shared her perspective on the importance of social participation:

*Before my retirement, my career brought me a great sense of satisfaction. Following retirement, I devoted ten years to studying calligraphy and painting at a university for seniors. However, since graduating, my social activities have significantly diminished as I’ve found myself mostly confined to staying at home. This change has honestly left me grappling with feelings of frustration and a sense of worthlessness.* [P21, Female, 85 years old]

***Older people’s needs for self-development***

**Digital Inclusion and Technology Use Training:** To stay connected, many older adults now seek to learn smartphone use, video calls, online shopping, and payment via WeChat or Alipay.

*My travel buddies and I often go on trips together—we usually sign up through the “Let’s Go Together” public WeChat account. Every time we visit a new place, we take photos. I’d really like to turn those photos into videos and share them online. I heard that teachers from the senior university sometimes come here to give classes, and I’d love to sign up for a video editing course if it’s available.* [P19, Female, 66 years old]

*At our Youzhu Elderly Service Station, meal assistance is currently available. However, placing an order requires responding in a WeChat group each day to select the desired set meal. For many of us older adults, this is quite challenging—our vision isn’t great, and we’re not familiar with using smartphones. I often end up missing the order, either because I respond too late or I reply to the wrong message thread. Zhang from the station has tried teaching me several times, but I just can’t seem to get the hang of it.* [P22, Female, 84 years old]

**File 5. The care problems that the SMART system was designed to diagnose and address**

| **Domains of care problems** | **Sub-domains of care problems** | **Items of care problems** |
| --- | --- | --- |
| 1. Decreased or lost self-care ability | 1. Risk for decreased or lost self-care ability | 1. Frailty, related to nutritional deficiencies |
|  |  | 2. Frailty, related to insufficient exercise |
|  |  | 3. Frailty, related to decreased balance and resistance |
|  | 2. Presence of decreased or lost self-care ability | 4. Decline in self-care ability |
|  |  | 5. Loss of self-care ability |
|  |  | 6. Self-care deficit in eating |
|  |  | 7. Decline in upper limb muscle strength |
|  |  | 8. Self-care deficit in tube feeding management |
|  |  | 9. Inability to cope with adverse reactions to tube feeding |
|  |  | 10. Self-care deficit in oral hygiene |
|  |  | 11. Self-care deficit in toilet hygiene |
|  |  | 12. Self-care deficit in dressing (upper and lower body) |
|  |  | 13. Self-care deficit in putting on/taking off footwear |
|  |  | 14. Self-care deficit in picking up objects |
|  |  | 15. Self-care deficit in bathing |
|  |  | 16. Self-care deficit in rolling left and right |
|  |  | 17. Inability to perform functional mobility from sitting on the side of the bed to lying down |
|  |  | 18. Decline in lower limb muscle strength |
|  |  | 19. Inability to perform functional mobility from lying down to sitting on the side of the bed |
|  |  | 20. Inability to perform functional mobility from sitting to standing |
|  |  | 21.  Inability to perform bed-to-chair/chair-to-chair transfers |
|  |  | 22. Decline in ability to answer and make phone calls |
|  |  | 23. Loss of ability to answer and make phone calls |
|  |  | 24. Self-care deficit in medication management |
|  |  | 25. Decline in ability to prepare light meals |
|  |  | 26. Decline in ability to perform household tasks |
|  |  | 27. Decline in ability to use public transportation |
| 2. Falls | 3. Risk for falls | 28. Risk for falls, related to hypoglycemia |
|  |  | 29. Risk for falls, related to impaired vision |
|  |  | 30. Risk for falls, related to environmental hazards |
|  |  | 31. Risk for falls, related to impaired hearing |
|  |  | 32. Risk for falls, related to weak lower limb muscle strength |
|  |  | 33. Risk for falls, related to poor balance |
|  |  | 34. Risk for falls, related to postural hypotension |
|  |  | 35. Risk for falls, related to insufficient knowledge of fall prevention |
|  |  | 36. Risk for falls, related to polypharmacy |
|  |  | 37. Risk for falls, related to side effects of medications |
|  |  | 38. Risk for falls, related to inappropriate shoes |
|  |  | 39. Risk for falls, related to psychological factors |
|  |  | 40. Risk for falls, related to urolithiasis |
|  | 4. Presence of falls | 41. Falls |
|  |  | 42. Risk for aggravated injuries due to improper injury management after falls |
|  |  | 43. Lack of knowledge regarding assessing injuries after falls |
|  |  | 44. Risk for fractures, related to falls |
|  |  | 45. Risk for functional decline due to decreased physical activity after falls |
|  |  | 46. Colles fractures |
|  |  | 47. Soft tissue injuries |
|  |  | 48. Hip fractures |
| 3. Delirium | 5. Risk for delirium | 49. Risk for delirium |
|  |  | 50. Risk for delayed detection of delirium |
|  | 6. Presence of delirium | 51. Risk for delayed detection of delirium causes |
|  |  | 52. Risk for falling from bed |
|  |  | 53. Risk for tube detachment |
|  |  | 54. Risk for self-harm or harm to others |
|  |  | 55. Risk that family members cannot properly cope with delirium |
|  |  | 56. Risk for prolonged delirium |
| 4. Dysphagia | 7. Risk for dysphagia | 57. Risk for dysphagia |
|  | 8. Presence of dysphagia | 58. Dysphagia |
|  |  | 59. Risk for weight loss and malnutrition |
|  |  | 60. Family members’ inability to manage tube feeding |
|  |  | 61. Risk for aspiration |
| 5. Incontinence | 9. Risk for incontinence | 62. Risk for stress urinary incontinence, related to obesity |
|  |  | 63. Risk for stress urinary incontinence, related to coughing |
|  |  | 64. Decreased bladder function |
|  |  | 65. Urinary tract infection |
|  |  | 66. Unconscious urination |
|  |  | 67. Delayed access to the toilet for defecation |
|  |  | 68. Risk for fecal incontinence, related to the use of laxatives |
|  |  | 69. Diarrhea |
|  | 10. Presence of incontinence | 70. Stress urinary incontinence |
|  |  | 71. Real urinary incontinence |
|  |  | 72. Fecal incontinence |
|  |  | 73. Bowel stoma |
| 6. Constipation | 11. Risk for constipation | 74. Poor bowel habits |
|  |  | 75. Risk for constipation, related to poor diet and insufficient water intake |
|  |  | 76. Risk for constipation, related to insufficient physical activity |
|  | 12. Presence of constipation | 77. Constipation |
| 7. Urinary retention | 13. Risk for urinary retention | 78. Dysuria |
|  | 14. Presence of urinary retention | 79. Urinary retention |
| 8. Cognitive decline | 15. Risk for cognitive decline | 80. Risk for cognitive decline, related to poor diet |
|  |  | 81. Risk for cognitive decline, related to insufficient physical activity |
|  |  | 82. Reduced interpersonal communication |
|  | 16. Presence of cognitive decline | 83. Memory decline |
|  |  | 84. Potential safety hazards at home |
|  |  | 85. Impaired sense of direction |
|  |  | 86. Fluctuations in cognition |
|  |  | 87. Difficulty in verbal communication |
|  |  | 88. Neuropsychiatric symptoms: manifested as agitation, aggression, compulsive behavior, abnormal collection of things, etc. |
|  |  | 89. Family members’ inability to cope with abnormal behaviors of older adults (e.g., excitement, irritability, hostility, and violent behavior) |
|  |  | 90. Family members’ ineffective response to caregiving burden |
|  |  | 91. Inability to self-eating, related to cognitive decline |
|  |  | 92. Inability to self-dressing, related to cognitive decline |
|  |  | 93. Risk for infections |
|  |  | 94. Lack of knowledge regarding drug therapy |
|  |  | 95. Cognition decline |
|  |  | 96. Inability to maintain personal hygiene |
| 9. Depression | 17. Risk for depression | 97. Risk for depression |
|  |  | 98. Negative emotion |
|  | 18. Presence of depression | 99. Depression |
|  |  | 100. Risk for suicide |
|  |  | 101. Side effects of antidepressants |
| 10. Impaired skin integrity | 19. Risk for impaired skin integrity | 102. Risk for impaired skin integrity, related to excessive sweating |
|  |  | 103. Risk for incontinence-associated dermatitis |
|  |  | 104. Risk for diabetic foot ulcers |
|  |  | 105. Risk for pressure ulcers, related to prolonged periods of unrelieved pressure |
|  |  | 106. Risk for improper pressure management |
|  |  | 107. Risk for pressure ulcers in the sacrococcygeal region, related to extended use of wheelchairs |
|  | 20. Presence of impaired skin integrity | 108. Impaired skin integrity: Stage 1 pressure ulcers |
|  |  | 109. Impaired skin integrity: Stage 2 pressure ulcers |
|  |  | 110. Impaired skin integrity: Stage 3 pressure ulcers |
|  |  | 111. Impaired skin integrity: Stage 4 pressure ulcers |
|  |  | 112. Incontinence-associated dermatitis |
|  |  | 113. Diabetic foot ulcers |
| 11. Common diseases | 21. Risk for common diseases | 114. Overweight |
|  |  | 115. High-sodium diet |
|  |  | 116. Excessive alcohol drinking |
|  |  | 117. Lack of knowledge regarding healthy diet |
|  |  | 118. Lack of knowledge regarding hypertension |
|  |  | 119. Smoking |
|  |  | 120. Unhealthy diet structure |
|  |  | 121. Insufficient physical exercise |
|  |  | 122. Lack of knowledge regarding diabetes mellitus |
|  |  | 123. Risk for diabetes mellitus |
|  |  | 124. Risk for myocardial infarction |
|  |  | 125. Risk for influenza |
|  |  | 126. Risk for pneumonia |
|  | 22. Presence of common diseases | 127. Poorly controlled blood pressure, related to unhealthy lifestyles |
|  |  | 128. Poorly controlled blood pressure, related to non-adherence to medication timing and dosage |
|  |  | 129. Poorly controlled blood pressure, related to irregular blood pressure monitoring |
|  |  | 130. Well-controlled blood pressure |
|  |  | 131. Positional hypotension, related to diagnosed diseases |
|  |  | 132. Positional hypotension, related to adverse drug effects |
|  |  | 133. Incorrect insulin usage, related to insufficient knowledge |
|  |  | 134. Hypoglycemia |
|  |  | 135. Well-controlled blood sugar |
|  |  | 136. Poorly controlled blood sugar, related to unhealthy lifestyles |
|  |  | 137. Poorly controlled blood sugar, related to nonadherence to medication timing and dosage |
|  |  | 138. Poorly controlled blood sugar, related to irregular blood sugar monitoring |
| 12. Psychosocial issues | 23. Risk of psychosocial issues | 139. Digital divide |
|  | 24. Presence of psychosocial issues | 140. Loneliness |
|  |  | 141. Social isolation |

**File 6. Suggestions on the evidence-based interventions and SMART system from the consultations with multidisciplinary expert panels**

| **Themes** |  | **Suggestions** |
| --- | --- | --- |
| **Round one and round two** | |  |
| Suggestions on the evidence-based | Balance function | 1. Organic diseases of the brain can also lead to decreased balance function, so the participation of experts from the neurology department and rehabilitation center is supposed to be involved in |
| interventions |  | 1. Use objective tools to evaluate older people’s balance function |
|  | Fall | 1. Advise older people to choose shoes without laces |
|  |  | 1. Some older people fall because of sudden syncope. However, the causes of syncope are too complex. We can select some major and changeable factors to further guide rehabilitation |
|  |  | 1. Consider falls due to delirium |
|  | Diet care | 1. Provide personalized diet guidance for different diseases, such as kidney diseases, diabetes mellitus, etc. |
|  | Nasal gastric tube feeding | 1. The temperature of the nutrient solution should be appropriate for the esophagus and stomach |
|  |  | 1. The most common problems of nasal gastric tube feeding at home are infection and stomach discomfort |
|  |  | 1. Proportion of nutrient solution should also be concerned |
|  | Strength training for lower limbs | 1. The usage and benefits of mobility devices and aids should also be delivered |
|  | Pneumonia management | 1. Add preventions and commonly used antibiotics for pneumonia caused by different pathogenic bacteria |
|  | Depression | 1. Improve depression by positive guidance and interpersonal communication |
|  |  | 1. Increase non-drug treatment for depression |
|  | Incontinence | 1. Explore different causes of cough carefully and perform cause-specific treatment |
|  | Disease management | 1. Pay attention to simple methods to assess diabetes foot, such as assessing dorsal foot artery pulsation and sensory nerve endings |
| Suggestions on the | Information collection | 1. Use voice and video to collect information intelligently and effectively |
| SMART system |  | 1. The information should be collected from simple problems to complex questions |
|  | Efficiency | 1. Because pictures taken by mobile phones cannot meet the requirements of vision assessment and screening of cataracts and glaucoma, we should advise older people to take pictures in professional places in the delivered interventions |
|  | Privacy | 1. Consider the privacy of older people when delivering tailored interventions |
|  | Feedback | 1. Avoid using the camera function of mobile phones too frequently for feedback |
|  | Compliance | 1. Make the delivered interventions as simple and clear as possible |
|  |  | 1. Use large bold text to deliver interventions |
|  |  | 1. Connect wearable devices to the SMART system to improve the compliance of older people |
| **Round three** |  |  |
| Suggestions | Cognitive | 1. Decline in cognitive function can be prevented |
| on the | screening | 1. Integrate cognitive training into rehabilitation |
| evidence-based | Oral care | 1. Oral hygiene is not the main problem for older people who cannot take care of themselves |
| interventions | Dressing | 1. Provide suggestions on the selection of suitable clothes and wearing and taking off skills for older people who have been in bed for a long time |
|  | Exercise | 1. Increase the amount of exercise according to the guidelines gradually |
| Suggestions on the SMART | Fall | 1. Instead of open-ended questions, possible options should be provided when asking older people about the causes of their falls, because they may not know the causes of falls |
| system | Oral care and dressing | 1. Provide suggestions about oral care and dressing skills through pictures |
|  | Evaluation tools | 1. Delete overlapping and unnecessary parts of different evaluation tools |
|  | Compliance | 1. Display older people’s performance ranking among their peers in the SMART system by histogram or the gap between peer ranking and achievement of their objectives |
|  |  | 1. Give real-time feedback to the older people |
|  |  | 1. Praise older people when they complete tailored interventions |
|  |  | 1. Don’t overwhelm older people with too much information every day |
|  | Effectiveness | 1. Update the contents involved in the SMART system |
|  | User-friendly interface | 1. The interface of the application should be displayed in different modules |
|  |  | 1. Recommendations should be displayed in different colors and sizes |
| **Round four** |  |  |
| Suggestions  on the  evidence- | Multimorbidity management | 1. Develop coordinated care strategies for older adults with multiple chronic conditions, prioritizing interventions to avoid duplication and ensure coherence. |
| based interventions | Sleep health support | 1. Incorporate non-pharmacological approaches for managing insomnia and circadian rhythm disturbances commonly observed in older populations. |
|  | Chronic pain management | 1. Provide structured assessment tools and a combination of pharmacological and non-pharmacological interventions to manage long-term pain. |
|  | Psychosocial issues | 1. Leverage large language models (e.g., ChatGPT, DeepSeek) to provide real-time guidance, personalized explanations, and interactive learning pathways for technology use. |
|  |  | 1. Offer interest-based digital literacy modules (e.g., video calling, online shopping, wearable devices) with step-by-step, visual instructions. |
| Suggestions on the | Dynamic personalization | 1. Enable adaptive care plans that evolve based on user feedback, changing health conditions, and preferences. |
| SMART  system | Scenario-based learning modules | 1. Provide brief, interactive videos simulating real-life situations to improve understanding of health behaviors and caregiving skills. |
|  | Emotional health monitoring | 1. Include mood check-ins or short emotional self-assessments to support mental health and guide appropriate referrals. |
|  | Adaptive delivery methods | 1. Use quick questions to identify adherence barriers (e.g., memory issues, confusion) and automatically adjust content delivery methods. |
|  | Motivational strategies and positive reinforcement | 1. Apply behavioral nudges such as progress badges, peer comparison charts, and real-time encouragement to foster long-term engagement. |
| **Round five** |  |  |
| Suggestions  on the  evidence-based interventions | Falls | 1. Integrate fall-risk prediction models into the SMART system using real-time data from wearable devices, environmental sensors, and medical history. Pair this with routine physical assessments and home modification strategies led by physiotherapists. |
|  |  | 1. Conduct regular home safety assessments to remove fall hazards (e.g., slippery rugs, poor lighting) and provide personalized recommendations for modifications. |
|  | Decreased or lost self-care ability | 1. Implement adaptive equipment (e.g., grab bars, shower chairs) and engage in one-on-one occupational therapy to help individuals regain or retain daily functional abilities. |
|  | Impaired skin integrity | 1. Use pressure-mapping sensors integrated into beds or chairs to detect early signs of pressure ulcers. Pair this with a regular repositioning schedule, personalized skin care routines. |
| Suggestions on the | Multilingual support | 1. Incorporate multiple languages and regional dialects to improve accessibility for diverse older populations. |
| SMART  system | Caregiver access interface | 1. Develop a parallel interface for family caregivers and community nurses to support real-time monitoring and coordinated care delivery. |
|  | Integration with services | 1. Link the SMART system to local health and social services to recommend nearby resources automatically. |
|  | Progress visualization over time | 1. Include time-series visualizations of key health metrics to help users track their progress and maintain motivation. |
